# Immune checkpoint inhibitor-related arthritis and oral disorders: shared pathophysiology and clinical implications for rheumatology and oral health

**DOI:** 10.1186/s12935-025-04040-1

**Published:** 2025-11-14

**Authors:** Mitra Abbasifard, Mobina Taghipoor, Mahsa Kimiae Talab, Hossein Khorramdelazad

**Affiliations:** 1 Department of Internal Medicine, Ali-Ibn Abi-Talib Hospital, School of Medicine, Rafsanjan University of Medical Sciences, Rafsanjan, Iran; 2 Student Research Committee, School of DentistryShiraz University of Medical Sciences, Shiraz, Iran; 3 Department of Oral and Maxillofacial Surgery, School of Dentistry, Rafsanjan University of Medical Sciences, Rafsanjan, Iran; 4 Department of Immunology, School of Medicine, Rafsanjan University of Medical Sciences, Rafsanjan, Iran

**Keywords:** ICI therapy, Arthritis, Oral diseases, Cancer immunotherapy

## Abstract

Immune checkpoint inhibitors (ICIs) have revolutionized contemporary cancer treatment by enhancing anti-tumor immune responses and mitigating the suppressive effects of inhibitory molecules within the tumor microenvironment (TME). However, these immunotherapies may also induce immune-related adverse events (IrAEs), resulting in various disorders, including arthritis and oral diseases, which can negatively impact patients’ quality of life and treatment outcomes. To effectively oversee ICI therapies and address immune dysregulation, it is essential to comprehend the interactions between these components. This review offers a comprehensive overview of ICI-induced arthritis and oral disorders, encompassing mechanisms, clinical presentation, diagnosis, and treatment strategies. Furthermore, it addresses emerging research pathways to improve clinical outcomes and patient care.

## Introduction

Immune checkpoint inhibitors (ICIs) have transformed cancer treatment by utilizing the immune system to attack tumors, resulting in sustained remissions in cancers such as melanoma, lung cancer, and renal cell carcinoma [1, 2]. These monoclonal antibodies (mAbs) specifically target inhibitory proteins, including cytotoxic T-lymphocyte antigen-4 (CTLA-4), programmed cell death protein-1 (PD-1), and programmed death-ligand 1 (PD-L1), thereby augmenting T-cell activation and anti-tumor immunity [3]. Following the inhibition, extensive immune activation may induce immune-related adverse events (irAEs) in 60–70% of patients, varying from moderate to life-threatening conditions [4]. Rheumatologic irAEs, such as inflammatory arthritis, and oral irAEs, including stomatitis, xerostomia, mucositis, and periodontitis, pose considerable challenges in diagnosis and treatment, potentially requiring treatment cessation and jeopardizing oncological outcomes [5, 6].

ICI-induced inflammatory arthritis (ICI-IA) impacts 4–6% of patients, with incidence ranging from 1% to 43% based on the type of ICI (greater with combinations), cancer type, and patient characteristics [7]. Clinically, it mimics rheumatoid arthritis (RA), spondyloarthropathies, or polymyalgia rheumatica, frequently manifesting as seronegative and asymmetric, distinguished by synovitis, tenosynovitis, or enthesitis. The pathophysiology arises from dysregulated T-cell responses, specifically CD8^+^ tissue-resident memory T cells, which are heightened in individuals with a history of osteoarthritis (OA) [8, 9]. Pro-inflammatory cytokines, such as interleukin (IL)−6, tumor necrosis factor-alpha (TNF-α), and interferon-gamma (IFN-γ), propagate this inflammation, resulting in chronic arthritis in 25% of instances even following the cessation of ICIs [10, 11]. ICI-IA may be associated with improved tumor responses, indicating shared immune activation mechanisms, but it entails a considerable disease burden [12]. Simultaneously, oral irAEs manifest in 5–7% of patients undergoing ICI therapy, encompassing xerostomia (5%), stomatitis/mucositis (up to 10%), dysgeusia, dysphagia, and periodontitis (4.7%, double the incidence in non-users) [6, 13, 14]. These result from immune-mediated malfunction of the salivary glands (sicca-like disease) and mucosal inflammation, resembling Sjögren’s syndrome or lichenoid reactions. The fundamental mechanisms include the production of autoantibodies, T-cell infiltration, and cytokine dysregulation, which compromise oral homeostasis and elevate the susceptibility to infection [15, 16].

ICIs induce both arthritis and oral disorders through intersecting immune pathways; these conditions exhibit bidirectional relationships, as recognized by the correlations between RA and periodontal disease (PD), where common cytokines (e.g., IL-6, IL-1, TNF-α) and citrullination mediated by *Porphyromonas gingivalis* facilitate mutual aggravation. This interaction creates a feedback loop in patients undergoing ICI treatment, potentially triggering irAEs and affecting cancer progression [17–22]. For instance, PD doubles the risk of RA, while patients with RA exhibit increased pulpal-periapical pathology [21].

Notwithstanding progress, this interaction in immunotherapy remains inadequately investigated, affecting diagnosis (e.g., differentiating between the impacts of chemotherapy and immunotherapy) and treatment (e.g., the effectiveness of methotrexate in ICI-IA, alongside the hazards associated with oral infections) [23, 24]. Significant deficiencies encompass inadequate integration of shared immunopathology, diagnostic biomarkers, and cohesive therapeutic alternatives. This discrepancy hinders personalized care, as irAEs may necessitate the cessation of ICIs, thereby worsening the prognosis [25, 26].

This study aims to provide a comprehensive synthesis of arthritis and oral disorders induced by ICIs, encompassing their pathophysiological underpinnings, clinical characteristics, diagnostic techniques, and therapeutic approaches. Furthermore, it highlights promising research avenues to optimize therapy outcomes and enhance patient care.

## Methodology

**Fig. 1:**
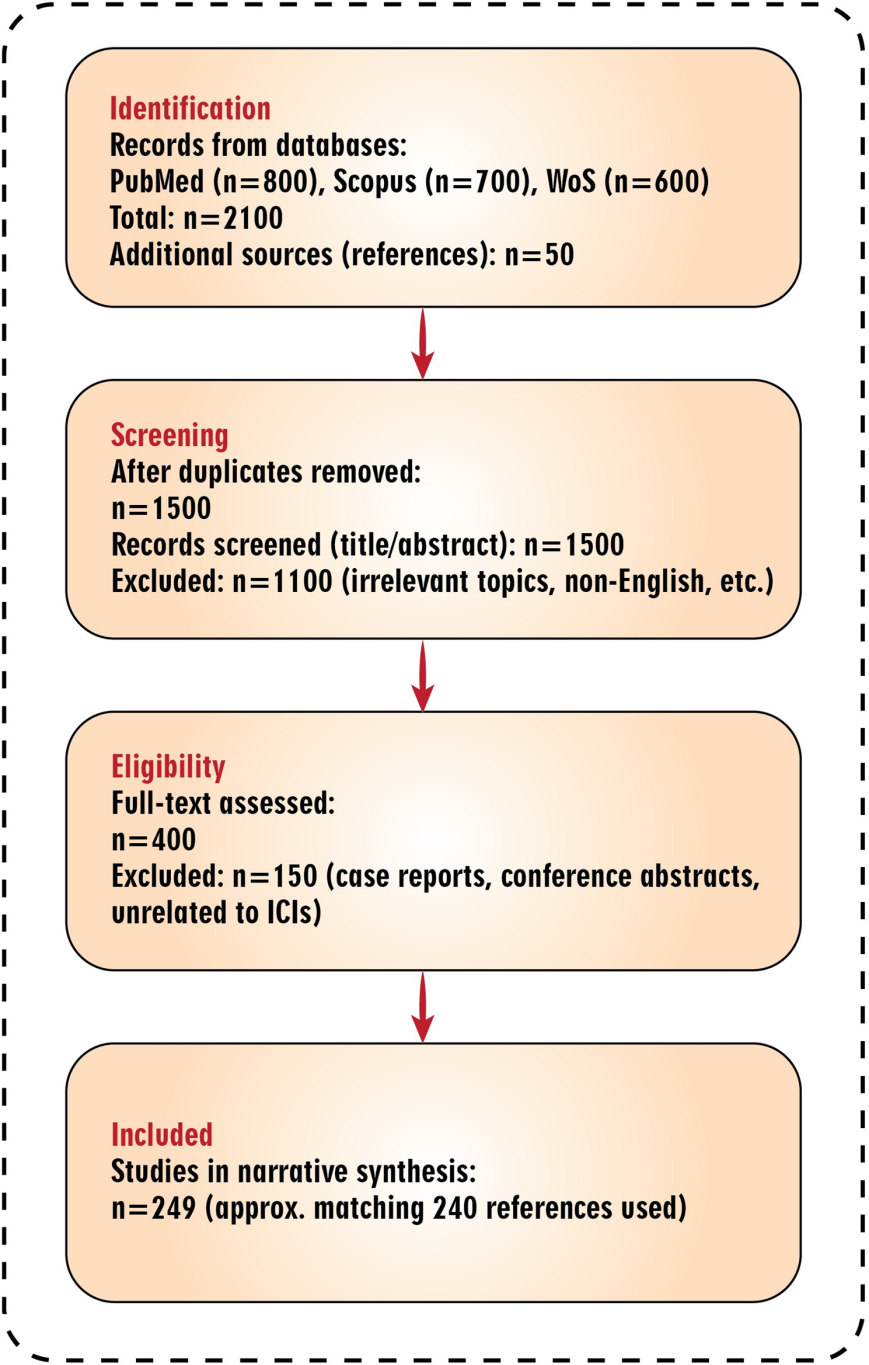
PRISMA flow diagram for the narrative review process. The diagram illustrates the structured literature search and selection of studies for the narrative synthesis. Databases searched included PubMed, Scopus, and Web of Science up to April 2025. Additional sources were identified from reference lists. Duplicates were removed using manual review. Screening was based on titles and abstracts for relevance to ICIs, arthritis, and oral disorders. Full-text assessment excluded non-peer-reviewed items, case reports, and unrelated studies. The final included studies (*n* = 249) form the basis of the narrative review, closely aligning with the 240 references cited

We performed a structured literature search with the PubMed, Scopus, and Web of Science databases to explore potential associations between ICIs, arthritis, and oral disorders. Only articles published by April 2025 were included. Search terms in the PubMed database included combinations of the following: “immune checkpoint inhibitors,” “arthritis,” “oral disorders,” “immune-related adverse events,” “autoimmunity,” “rheumatic diseases,” and “cancer immunotherapy.” The inclusion criteria included original research articles, systematic reviews, or meta-analyses published in peer-reviewed journals written in English that focused on the mechanisms, clinical manifestations, or management of ICIs, arthritis, and oral disorders. Exclusion criteria included non-peer-reviewed articles, conference abstracts, studies unrelated to ICIs and related adverse effects, and duplicate publications in multiple databases. Images, titles, and abstracts were reviewed for relevance; eligible articles underwent full-text review. Two independent reviewers screened the studies for inclusion, reaching consensus through discussion. We extracted and narratively synthesized data relevant to mechanisms, clinical manifestations, and management strategies to identify knowledge gaps and propose avenues for future investigation. The study selection process is illustrated in the PRISMA flow diagram (Fig. 1), which has been adapted for this narrative review to enhance transparency and clarity. A summary of inclusion and exclusion criteria is provided in Table 1. Although this is a narrative review rather than a systematic one, we employed a structured search to identify approximately 2100 records, leading to 249 studies included in the synthesis (closely matching the 240 cited references).

**Table 1:** Summary of inclusion and exclusion criteria for study selection

Category	Criteria details
**Inclusion criteria**	• Original research articles, systematic reviews, or meta-analyses. • Published in peer-reviewed journals in English. • Focused on mechanisms, clinical manifestations, or management of ICIs, arthritis, and oral disorders. • Published by April 2025. • Relevant to associations between ICIs and irAEs.
**Exclusion criteria**	• Non-peer-reviewed articles (e.g., gray literature). • Studies unrelated to ICIs and related adverse effects. • Duplicate publications across databases. • Conference abstracts • Non-English publications or those lacking full-text access.

## Immune checkpoint inhibitors in cancer therapy

**Fig. 2:**
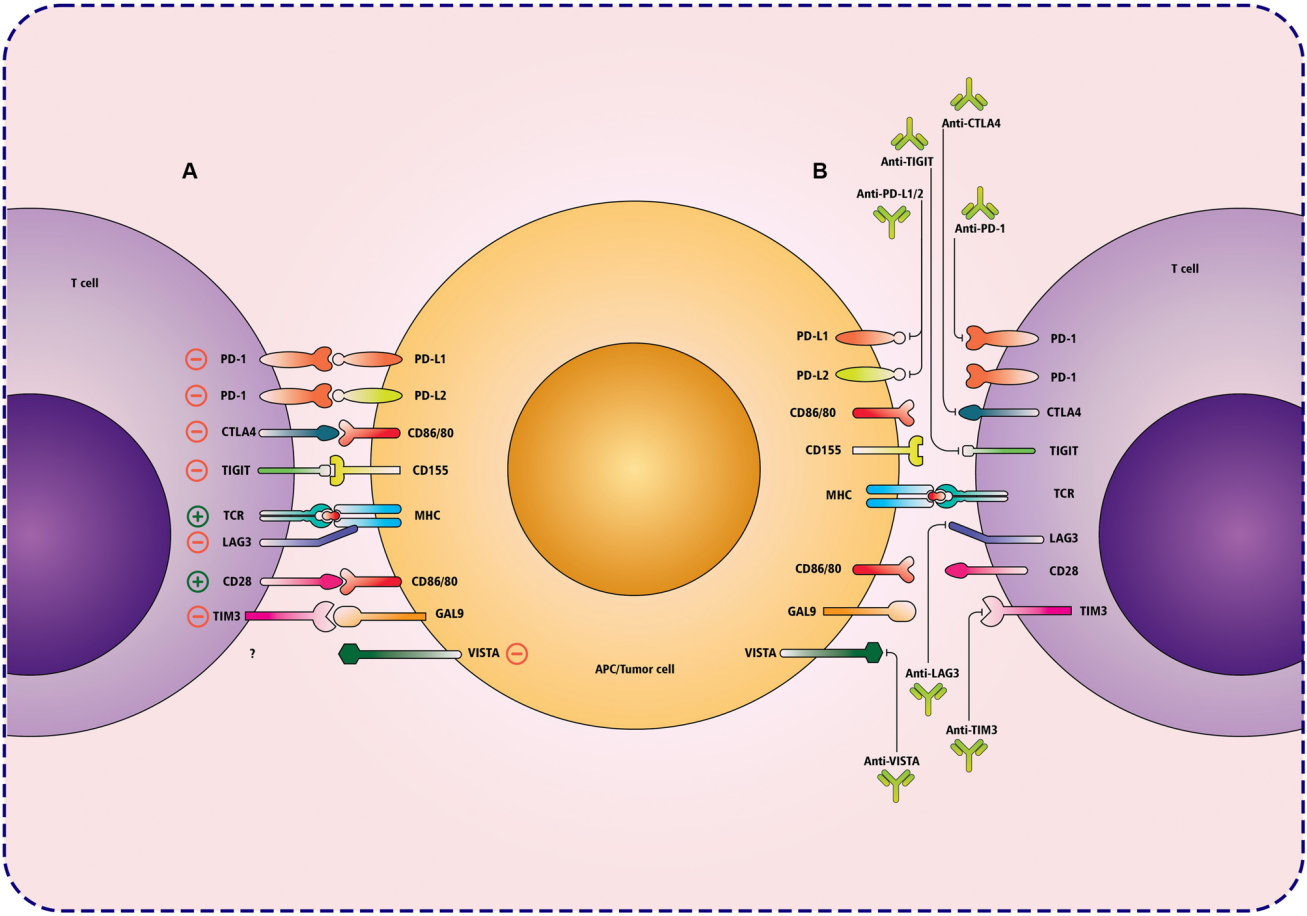
Mechanisms of T cell inhibition by APC/tumor cells and corresponding immune checkpoint blockade therapies. The interactions between immune checkpoint molecules on T cells and APCs or tumor cells highlight inhibitory and stimulatory pathways. **(A)** Various immune checkpoint molecules, including PD-1, PD-L1, PD-L2, CTLA-4, TIGIT, LAG3, TIM-3, and VISTA, interact with their respective ligands on APCs or tumor cells, leading to T cell exhaustion and immune evasion within the TME. Additionally, co-stimulatory molecules such as CD28 engage with CD86/CD80 to provide necessary activation signals for T cell proliferation and effector function. (**B**) ICI therapy involves monoclonal antibodies targeting inhibitory pathways, including PD-1/PD-L1, CTLA-4, TIGIT, TIM-3, LAG3, and VISTA, thereby restoring T cell effector function and enhancing anti-tumor immunity. These therapeutic interventions prevent immune suppression, enabling T cells to recognize and eliminate cancer cells more effectively. PD-1; programmed cell death 1, PD-L1; programmed death-ligand 1, PD-L2; programmed death-ligand 2, CTLA-4; cytotoxic T-lymphocyte-associated protein 4, TIGIT; T cell immunoreceptor with Ig and ITIM domains, LAG3; lymphocyte activation gene 3, TIM-3; T cell immunoglobulin and mucin-domain containing-3, VISTA; V-domain Ig suppressor of T cell activation, APC; antigen-presenting cell, TCR; T cell receptor, MHC; major histocompatibility complex, GAL9; galectin-9

Cancer treatments by ICIs have been associated with promising clinical outcomes. These mAbs markedly enhance survival rates and elicit sustained anti-tumor responses in resistant malignancies [27]. The U.S. Food and Drug Administration (FDA) has approved several ICIs that target PD-1, PD-L1, and CTLA-4 for various cancers [28]. In addition to these, mAbs targeting novel checkpoints, including lymphocyte activation gene 3 (LAG-3), T-cell immunoglobulin and mucin-domain containing-3 (TIM-3), T-cell immunoreceptor with Ig and ITIM domains (TIGIT), and V-domain Ig suppressor of T-cell activation (VISTA), are currently undergoing clinical evaluation [29–32] (Fig. 2). These ICIs aim to enhance immune recognition and the eradication of tumor cells by suppressing tumor growth, proliferation, and immune evasion. Clinical trials are assessing the safety, effectiveness, and value of monotherapies and combination therapies [33]. Although these therapies enhance anti-tumor immunity, they may compromise immune tolerance, resulting in irAEs such as arthritis and oral disorders, which present as inflammatory or autoimmune-like conditions affecting joints, mucocutaneous tissues, and other organs [34, 35]. Understanding these associations is crucial for improving patient care in populations treated with ICIs.

### Shared mechanisms of established ICIs (Integrating PD-1/PD-L1 and CTLA-4 pathways)

PD-1 and PD-L1 checkpoints suppress T-cell activation and anti-tumor responses, while CTLA-4 inhibits initial T-cell activation in lymph nodes by competing with CD28 for B7 ligands on antigen-presenting cells (APCs), thereby fostering anergy and enhancing the function of Tregs [36–39]. Anti-PD-1/PD-L1 mAbs, such as pembrolizumab, nivolumab, and cemiplimab (anti-PD-1), along with atezolizumab, avelumab, and durvalumab (anti-PD-L1), inhibit this pathway to augment CD8^+^ T-cell function, resulting in tumor regression in melanoma, renal cell carcinoma, non-small cell lung cancer (NSCLC), and bladder cancer; similarly, ipilimumab, an anti-CTLA-4 mAb, improves outcomes in melanoma and hepatocellular carcinoma (HCC) by activating T-cell responses and reducing immunosuppressive components such as Tregs and myeloid-derived suppressor cells (MDSCs) [40–46]. These shared inhibitory mechanisms, when blocked, disrupt immunohomeostasis, akin to autoimmune disorders, affecting areas such as the skin, gastrointestinal tract, liver, lungs, mucocutaneous tissues (including oral mucosa), and endocrine system, with arthritis occurring from unregulated T-cell activation against joint tissues and oral disorders like mucositis or sicca-like symptoms stemming from mucosal inflammation [34, 35, 47].

For instance, combining chemotherapy with additional ICIs frequently enhances outcomes, but this broad immune activation often triggers irAEs in 60–70% of patients, ranging from mild to life-threatening, with higher rates observed in combination therapies involving PD-1/PD-L1 and CTLA-4 inhibitors [4, 43, 48–50]. Notably, an increased prevalence of irAEs may correlate with improved treatment outcomes, reflecting common immune activation pathways, though it imposes a significant disease burden and necessitates diligent monitoring [12, 35, 46]. These converging findings from multiple studies highlight the trade-off between efficacy and toxicity, where CTLA-4 inhibition undermines self-tolerance more profoundly than PD-1/PD-L1 blockade, elevating the likelihood of autoimmune-like conditions in joints (arthritis) and mucocutaneous regions (oral sores), as evidenced by case reports of severe irAEs like grade 4 mucositis and esophagitis associated with pembrolizumab [44, 45, 47].

### Emerging inhibitory ICIs (LAG-3, TIM-3, TIGIT, VISTA)

LAG-3 regulates T-cell responses through major histocompatibility (MHC) class II interaction, facilitating tumor immune evasion similar to PD-1, while anti-LAG-3 medicines like ieramilimab demonstrate limited efficacy as monotherapy but exhibit enhanced responses (e.g., 10.7% overall response rate [ORR]) when combined with anti-PD-1 mAbs such as spartalizumab in advanced solid tumors. Treatment-related adverse events (TRAEs) encompass fatigue, nausea, and irAEs such as colitis and polyarthritis, with combination therapy increasing the possibility of grade 3/4 occurrences [51–53]. In comparison to PD-1/PD-L1 inhibitors, LAG-3 inhibition may increase the risk of arthritis due to enhanced T-cell dysregulation, whereas oral conditions may arise from extensive mucosal inflammation, highlighting the necessity for combination-specific irAE profiling; LAG-3 serves a complex function in immune modulation, exhibiting context-dependent effects that are especially pertinent in autoimmune conditions, where in mouse models of autoimmune diabetes. LAG-3 targeting restricts the proliferation and suppressive capabilities of Tregs, potentially exacerbating autoimmunity.

In contrast, in individuals with RA, LAG-3⁺ Tregs are prevalent in inflamed regions and secrete significant quantities of IL-10, indicating an anti-inflammatory role [53–56]. TIM-3 facilitates apoptosis in Th1 cells through galectin-9 and is present on dendritic cells (DCs) and natural killer (NK) cells, with anti-TIM-3 mAbs, such as sabatolimab, exhibiting minimal efficacy as monotherapy but showing improved effectiveness (6% partial response rate) when combined with anti-PD-1 agents in solid tumors, including NSCLC and colorectal cancer. Frequent TRAEs, such as tiredness, occur more commonly in combination therapies. In contrast to CTLA-4 inhibitors, TIM-3 inhibition may offer reduced risks of arthritis but comparable potential for oral irAEs due to innate immune disruption [57–60]. TIM-3 is also expressed on NK cells (25–97% in healthy donors), with an increased mean fluorescence intensity (MFI) upon stimulation by tumor cells, potentially contributing to NK cell exhaustion in cancers such as gastric adenocarcinoma, necessitating focused evaluations in high-risk populations [61].

TIGIT engages with CD155/CD112 on tumor cells and APCs, inhibiting T-cell activity, and anti-TIGIT mAbs like vibostolimab demonstrate modest ORRs of 5–7% either as monotherapy or in combination with pembrolizumab in anti-PD-1-refractory NSCLC. Regarding TRAEs, including pruritus and fatigue, this approach may aggravate arthritis by heightened TIL infiltration and joint inflammation. In contrast, oral effects may resemble those of PD-1 inhibitors, indicating shared irAE pathways that necessitate comparative investigations [56, 62]. In addition to its function in inhibiting T-cell activity, TIGIT is expressed on NK cells, where it regulates cytotoxicity and facilitates immune evasion in malignancies, with TIGIT present on 25–97% of circulating NK cells in healthy donors and exhibiting a greater MFI on mature CD56dim NK cells; co-incubation with tumor cells elevates TIGIT MFI by roughly 56%, and in gastric cancer patients, TIGIT expression on NK cells is not significantly different from that in healthy controls, suggesting that TIGIT may be a potential dual target for T and NK cell-based immunotherapies that could enhance anti-tumor responses, particularly when combined with PD-1 inhibitors, and potentially mitigate irAEs like arthritis by increasing NK cell infiltration in joints [61, 63].

VISTA, a member of the B7 family, suppresses T-cell activity through P-selectin glycoprotein ligand-1 (PSGL-1)/V-Set and Immunoglobulin domain-containing 3 (VSIG-3) and stimulates cytokine secretion in macrophages. Anti-VISTA mAbs, such as CI-8993, exhibit controllable pharmacokinetics and immune activation in solid tumors, accompanied by temporary elevations in cytokines. The preliminary assessment of CI-8993 demonstrated pharmacodynamic profiles indicative of both innate and adaptive immune activation, characterized by temporary cytokine elevations and alterations in monocyte and T-cell activation markers, suggesting a pro-inflammatory reprogramming [64–68]. The absence of dose-limiting toxicities, combined with the reported pharmacokinetic profile, supports ongoing development. Nonetheless, the absence of a definitive dose-response for critical effector cytokines, such as TNF-α and CCL4, highlights the intricacy of VISTA control, establishing CI-8993 as a promising candidate for combination therapies, especially in cold tumors characterized by myeloid-driven suppression [68].

Moreover, VISTA enhances innate immune responses in autoimmune contexts, and preclinical investigations utilizing the collagen antibody-induced arthritis (CAIA) model have demonstrated that genetic ablation or pharmacological inhibition of VISTA results in reduced joint inflammation, decreased C5a receptor signaling on myeloid cells, and enhanced expression of anti-inflammatory mediators, including IL-1 receptor antagonist [69]. Furthermore, VISTA expression in human synovial tissue indicates possible translational significance for RA; these outcomes have significant implications for cancer immunotherapy, as VISTA blockade aims to rejuvenate exhausted T cells in the TME. However, its inhibition may simultaneously diminish harmful myeloid-driven inflammation in autoimmune disorders, contrasting with PD-1 and CTLA-4 inhibitors, which frequently aggravate or induce autoimmune symptoms [70, 71]. Consequently, VISTA-targeted treatments may provide therapeutic benefits by boosting antitumor immunity without exacerbating, and potentially improving, autoimmune disorders such as RA. However, the dual functions of VISTA within immunological compartments necessitate further assessment in clinical studies, particularly in individuals with coexisting autoimmunity [69–71].

### Bispecific antibodies (BsAbs)

In recent years, bispecific antibodies (BsAbs) have emerged as an innovative approach in tumor immunotherapy, integrating two separate antigen targets within a single antibody molecule to improve therapeutic efficacy and safety, thereby accelerating the advancement and widespread utilization of BsAbs [72]. PD-L1/TIGIT nanobodies augment T-cell activation by 40% in vitro, while VISTA/PD-L1 BsAbs promote enhanced cancer cell lysis (40–50%) through effector cytokines, and polymeric PD-1/PD-L1 BsAbs attain 90% tumor suppression in preclinical studies [73–76]. Cadonilimab, a BsAb that targets PD-1 and CTLA-4, regulates T-cell activity to boost anti-tumor immune responses. This dual checkpoint blockade can compromise peripheral immunological tolerance, thereby heightening the likelihood of irAEs, particularly those affecting the oral mucosa. The etiology of oral lichenoid lesions is likely to involve T-cell-mediated cytotoxicity and localized immunological dysregulation [77].

The biopsy indicated infiltration by CD4^+^ and CD8^+^ T cells, B cells (CD20^+^), macrophages (CD68^+^), and myofibroblasts (α-SMA^+^), implying a vigorous immune-mediated inflammatory response [77]. These immune cells can attack basal keratinocytes and subepithelial tissues, resulting in lichenoid tissue damage and mucosal erosion. The presence of myofibroblasts may signify continuous tissue remodeling or fibrosis resulting from chronic inflammation. Accordingly, cadonilimab can induce considerable oral mucosal toxicity, likely via mechanisms akin to other ICIs, but exacerbated by its dual-target mechanism, making timely identification and immunosuppressive therapy essential for addressing these challenges [77]. The dual inhibition of PD-L1 and LAG-3 using the BsAb IBI323 enhances T-cell-mediated anti-tumor responses beyond the efficacy of monotherapies or combination therapies, indicating a synergistic mechanism of immune activation. The antibody’s capacity to bind PD-L1⁺ and LAG-3⁺ cells may promote the spatial organization of immune effector functions within the TME, leading to increased cytokine production and the clonal growth of tumor-specific T cells [78]. These findings challenge the traditional notion that co-inhibitory receptor blockage requires separate targeting, instead highlighting the therapeutic potential of multifunctional agents in overcoming acquired resistance to ICIs. Moreover, PD-L1/LAG-3 BsAbs, such as IBI323, achieve 75% inhibition, accompanied by an increase in tumor-infiltrating lymphocytes (TILs). In combination with TGF-β/PD-L1 BsAbs, Mn^2+^ enhances tumor shrinkage by 80% through STING activation [78, 79]. In comparison to single-target ICIs, BsAbs may increase the risk of arthritis due to synergistic immune amplification. However, they could reduce oral irAEs by targeted TME modulation, subject to clinical confirmation [78, 79] (Table 2).

**Table 2:** ICI therapies’ findings in various cancers

Target/Agent	Immunologic function	Clinical application & efficacy	irAEs	Ref
**PD-1/PD-L1 inhibitors**	Blocks PD-1/PD-L1 to restore cytotoxic T-cell responses	Approved for melanoma, NSCLC, RCC, bladder cancer; shown to induce tumor regression and improve survival [e.g., pembrolizumab, nivolumab]	irAEs include arthritis, mucositis, xerostomia, pneumonitis, thyroiditis; Grade 4 mucositis reported with pembrolizumab in head and neck cancer	[40–43, 47]
**CTLA-4 (e.g.**,** ipilimumab)**	Enhances T-cell priming by inhibiting CTLA-4/B7 binding; reduces Treg-mediated suppression	Improves survival in melanoma and hepatocellular carcinoma	High risk of arthritis and oral ulcers; severity correlates with efficacy	[44–46]
**LAG-3 (e.g.**,** ieramilimab)**	Regulates T cells via MHC-II; modulates Treg and effector functions	Limited monotherapy effect; ORR = 10.7% when combined with spartalizumab in solid tumors	TRAEs include colitis, polyarthritis; mucosal inflammation may trigger oral irAEs	[51, 52]
**TIM-3 (e.g.**,** sabatolimab)**	Promotes Th1 apoptosis via galectin-9; expressed on DCs and NK cells	Low monotherapy efficacy; combination with PD-1 inhibitors yields ~ 6% PR in NSCLC and CRC	Increased fatigue and possible oral irAEs in combination regimens	[59, 60]
**TIGIT (e.g.**,** vibostolimab)**	Inhibits T cells via CD155/CD112 binding	5–7% ORR in PD-1-resistant NSCLC with or without pembrolizumab	TRAEs include pruritus, fatigue; arthritis risk due to joint infiltration by activated T cells	[56, 62]
**VISTA (e.g.**,** CI-8993)**	Modulates T-cell and myeloid activity via VSIG-3/PSGL-1	Phase 1 data shows immune activation, transient cytokine surge, no dose-limiting toxicity	May reduce arthritis in autoimmune models; oral irAEs not well-defined	[67, 68]
**PD-L1/TIGIT BsAb**	Dual checkpoint blockade enhances T-cell activation	In vitro: 40% increased T-cell activity	May heighten arthritis risk; oral irAEs unclear	[73, 74]
**VISTA/PD-L1 BsAb**	Blocks adaptive (PD-L1) and innate (VISTA) suppression	Induces 40–50% cancer cell lysis in vitro via cytokine release	Potential reduction in oral inflammation; arthritis risk TBD	[75]
**Polymeric PD-1/PD-L1 BsAb**	Dual inhibition with enhanced receptor binding avidity	Achieves 90% tumor suppression in preclinical models	High irAE potential; robust immune activation may lead to arthritis	[76]
**Cadonilimab (PD-1/CTLA-4 BsAb)**	Dual checkpoint blockade for enhanced anti-tumor T-cell response	Shown to improve tumor response; biopsy shows CD4+, CD8^+^, CD20^+^, CD68^+,^ α-SMA^+^ cell infiltration in oral lesions	Significant oral mucosal toxicity (lichenoid lesions), arthritis risk elevated due to T-cell hyperactivity	[77]
**IBI323 (PD-L1/LAG-3 BsAb)**	Simultaneous targeting facilitates spatial coordination of immune cells	Yields 75% tumor inhibition; increases TILs and cytokine levels	Arthritis risk increased; oral irAEs potentially reduced via targeted TME modulation	[78]
**Mn²⁺ + TGF-β/PD-L1 BsAb**	Activates STING pathway while blocking immunosuppressive PD-L1/TGF-β axis	80% tumor shrinkage observed in preclinical models	Autoimmune exacerbation (e.g., arthritis) possible via STING activation	[79]

Collectively, ICI therapy targeting PD-1/PD-L1, CTLA-4, LAG-3, TIM-3, TIGIT, and VISTA has advanced cancer immunotherapy, with combinations and BsAbs augmenting efficacy. Nonetheless, irAEs such as arthritis (due to joint inflammation), polymyalgia rheumatica, and oral disorders (stemming from mucosal or autoimmune effects) persist as significant obstacles, frequently associated with therapeutic response but necessitating individualized therapy [16, 64]. Future research should focus on identifying biomarkers for irAEs and developing approaches to reduce their incidence, thereby optimizing treatment benefits.

## Associations between ICIs and arthritis

This section addresses the underlying immunological mechanisms in ICI-induced arthritis and oral disorders. Furthermore, it emphasizes the emerging correlation between oral health conditions, particularly PERIODONTAL DISEASE, and arthritis. Understanding these mechanisms may open new avenues for the development of interdisciplinary approaches in immunopathology, oncology, rheumatology, and dentistry, thereby supporting improved management of these interconnected conditions (Table 3).

**Table 3:** Summary of mechanisms and clinical features of ICI-induced arthritis

Study type	Intervention	Study details	Proposed mechanisms	Key results and figures	Ref
Prospective Research Study	ICIs (PD-1/CTLA-4 inhibitors) for advanced malignancies	• Analysis of serum cytokines (IFN-γ, IL-6, TNF-α, VEGF-A, etc.) in 10 patients with ICI-induced inflammatory arthritis (ICI-IA) vs. 10 ICI-treated controls without IA • Demographics: Median age 62 years; cancers: Melanoma, NSCLC. • Assays: U-PLEX platform (Meso Scale Discovery). Adjusted for confounders (age, sex, steroid use).	• Elevated TNF-α and VEGF-A suggest pro-inflammatory cytokine dysregulation driving synovitis • Parallels RA pathogenesis with angiogenesis and immune activation. • No significant differences in other cytokines (e.g., IL-17, IFN-γ).	• Higher TNF-α (*p* = 0.02) and VEGF-A (*p* = 0.01) in ICI-IA vs. controls. • Ordinal logistic regression: TNF-α associated with worse CDAI (OR 1.5, 95% CI 1.1–2.0). • 70% of ICI-IA patients required steroids/DMARDs • No impact on cancer response noted.	[113]
Review	Anti-CTLA-4 (ipilimumab), anti-PD-1 (nivolumab, pembrolizumab), anti-PD-L1	• Literature review comparing ICI-IA to reactive arthritis (ReA); adults aged 30–80 s with even gender distribution; methods involve comparison to RA and SpA.	• Reactivation of autoreactive joint-specific T cells • Direct effects on monocytes promoting inflammatory cytokines, tissue extravasation, and osteoclast differentiation for CTLA-4 blockade • PD-1 blockade promotes autoreactive adaptive and pro-inflammatory innate immune responses.	• Prevalence of ICI-IA: 3–7.5.5%; 5.5% RF positive, 5% ACPA positive • 49% had persistent IA at 6 months post-ICI cessation • Treatments include hydroxychloroquine, sulfasalazine, methotrexate, leflunomide, TNF-inhibitors, IL-6 inhibitors • Rapid bone resorption and erosions within months reported.	[114]
Review (literature comparison of ICI-IA and ReA)	ICIs	• Included 21 studies on ICI-IA (prospective and retrospective) and 14 studies on ReA (retrospective, 1 RCT) • Focused on treatment with NSAIDs, GC, DMARDs • No specific patient numbers or demographics provided.	• Both ICI-IA and ReA characterized by synovial reaction to a well-defined causal event (ICI therapy for ICI-IA, infection for ReA).	• ICI-IA: Effective treatments include NSAIDs, GC, SSZ, MTX, HCQ, TNFi • Small case reports show IL-6Ri effects. • ReA: Effective treatments include NSAIDs, GC, SSZ (from RCT), MTX and SSZ with TNFi (retrospective); small case reports show IL-6Ri effects. • Similarities and differences in pathogenesis and clinical features noted • Potential for self-limiting disease course important for treatment strategy.	[116]
Retrospective Study	Pembrolizumab, nivolumab, cemiplimab, atezolizumab, avelumab, durvalumab, ipilimumab, tremilumab	• 89 ICI-IA cases out of 2451 cancer patients (March 2011-January 2021) at Northwestern University • Demographics showed no significant differences in sex, age, race, ethnicity; common cancers: lung (38%), melanoma (33%), kidney (23%); common ICIs: pembrolizumab (32%), nivolumab (26%), nivolumab + ipilimumab (30%) • Methods included EHR review, logistic regression, and random forest machine learning models.	• Not explicitly detailed.	• Incidence: 3.6% (89/2451) • Outcomes: 20% stopped ICI therapy due to arthritis, median onset 20 weeks post-ICI • Treatments: steroids (48%), NSAIDs (37%); statistics: melanoma (OR = 1.99, 95% CI 1.08–3.65) and renal cell carcinoma (OR = 2.03, 95% CI 1.06–3.84) more likely to develop ICI-IA vs. lung cancer; nivolumab + ipilimumab (OR = 1.86, 95% CI 1.01–3.43) vs. pembrolizumab • Higher odds of cutaneous (OR = 2.66, 95% CI 1.63–4.35), endocrine (OR = 2.09, 95% CI 1.15–3.80), and gastrointestinal irAEs (OR = 2.88, 95% CI 1.76–4.72) in ICI-IA patients vs. controls. Machine learning models achieved accuracies of 0.79 (logistic regression) and 0.80 (random forest).	[118]
Prospective Observational Study	ICIs (anti-PD-1/anti-CTLA-4, monotherapy/combination) for advanced cancers	• Longitudinal cohort of 35 patients with ICI-induced IA referred to rheumatology (June 2015–December 2018). • Follow-up: Median 9 months post-IA onset. Assessments: DAS28, tumor response (RECIST). • Cox models for persistence predictors.	• Persistent T-cell activation post-ICI cessation • Combination therapy amplifies immune dysregulation via dual checkpoint blockade, increasing cytokine storms (e.g., IL-6, TNF-α). Multiple IRAEs indicate broader immune overactivation.	• IA persisted >12 months in 50%; 90% required steroids, 40% csDMARDs/bDMARDs (e.g., MTX, TNF inhibitors). • Combination ICI: HR 2.5 for persistence (95% CI 1.2–5.1). No adverse impact on tumor response (*p* = 0.45 for DMARDs vs. progression).	[119]
Observational Case Series	Immune Checkpoint Inhibitors (ICIs) for malignancy (e.g., PD-1/PD-L1 inhibitors like nivolumab, pembrolizumab)	• Retrospective analysis of 8 patients with histologically confirmed malignancy treated with ICIs who developed exacerbated osteoarthritis (termed ICI-activated OA or IOA). • Patients assessed by rheumatologists at multiple centers (e.g., Hospital for Special Surgery, University of Chicago). Inclusion: Symptomatic worsening of OA post-ICI initiation. • Median age: Not specified; joints affected: Knees, hips, hands. • Timing: Onset 1–22 months post-ICI (most within 6 months).	• ICI blockade exacerbates underlying low-grade inflammation in OA via increased cytokine expression (e.g., IL-1β, TNF-α) and immune cell infiltration (e.g., macrophages, T-cells) in synovial tissue, promoting angiogenesis and joint damage. • Phenotypic variation in OA (inflammatory vs. non-inflammatory) may predispose to exacerbation.	• 5/8 patients experienced OA worsening after ICI cessation; treatments: Intra-articular steroids (most common), NSAIDs, physical therapy • No DMARDs required. Imaging: Ultrasound/X-ray showed osteophytes, effusions. Largest case series to date • Suggests need for vigilance in first 6 months. No cancer outcomes reported.	[120]
Systematic Review	ICIs targeting PD-1, CTLA-4, PD-L1 (e.g., nivolumab, ipilimumab, pembrolizumab)	• Systematic review of 52 studies (33 clinical trials, 3 observational, 16 case reports) on musculoskeletal/rheumatic IRAEs. • Search: Medline/Embase (up to 2016). • Outcomes: Arthralgia, arthritis, myalgia, sicca, vasculitis. Pooled from 1,725 unique records.	• ICIs disrupt immune tolerance, enhancing T-cell activation and cytokine release (e.g., TNF-α, IL-6), leading to autoimmunity mimicking RA, PMR, or vasculitis. • Preexisting autoimmunity may flare due to unchecked anti-tumor responses.	• Arthralgia: 1–43% incidence (24 trials); arthritis: 1–7% (5 trials); myalgia: 2–21% (12 trials). • Case reports: 9 inflammatory arthritis, 3 myositis, 2 vasculitis. • In preexisting autoimmunity: 30–50% flare rate. • No meta-analysis due to heterogeneity. Calls for standardized IRAE reporting.	[121]
Systematic Review	Anti-CTLA-4, anti-PD-1, anti-PD-L1 antibodies	• No specific number of cases/patients or demographics provided; focuses on current evidence and case series/reports.	• Increased autoreactive T cells, inflammatory cytokines (TNF-α, IL-6, IL-17), epitope spreading, prevention of B cell apoptosis leading to increased autoantibody loads.	• Incidence: 2% prevalence in cancer immunotherapy patients (retrospective cohort, 34 patients, mean age 59.2 years, 65% polyarticular, 35% oligoarticular, mean CRP 5.14 mg/dL, RF and CCP antibodies negative) • Outcomes: Treated with NSAIDs, analgesics, intra-articular/systemic glucocorticoids, csDMARDs (methotrexate, hydroxychloroquine, sulfasalazine), bDMARDs (TNF-α, IL-6 inhibitors) • Statistics: Anti-RA33 antibodies in 11.4% of ICI-induced arthritis patients vs. none in controls without arthritis.	[122]
Systematic Review, Case Series	Anti-CTLA-4 (e.g., ipilimumab), anti-PD-1 (e.g., pembrolizumab, nivolumab, cemiplimab), anti-PD-L1 (e.g., atezolizumab, avelumab, durvalumab), anti-LAG-3, anti-TIM-3, anti-TIGIT, anti-VISTA	• Number of cases/patients varies by study (e.g., cohort of 69 patients with arthritis during ICI treatment, 87 patients improved with GCs and anti-IL-6 receptor) • Demographics not specified; methods include clinical trials, in vitro, and animal models.	• Increased T-cell activity against antigens in tumors and healthy tissues, increased inflammatory cytokine levels, increased levels of preexisting autoantibodies, enhanced complement-mediated inflammation due to direct CTLA-4 binding on normal tissue.	• Incidence: ICI-induced arthralgia up to 43%, inflammatory arthritis ~ 7% • Outcomes: ICI-IA can persist post-therapy, causing irreversible joint damage • Treatments: NSAIDs, GCs, csDMARDs (e.g., methotrexate), bDMARDs (e.g., anti-TNF, anti-IL-6 receptor, anti-IL-17 A) • Statistics: 80% improvement with anti-TNF, 40% cancer remission • High GC doses (>7.5 mg prednisone daily) linked to reduced survival in melanoma patients.	[128]

Evidence reveals that rheumatic and systemic irAEs encompass a wide range of known rheumatic diseases [80], including arthralgia/arthritis, enthesitis, polymyalgia rheumatica (PMR), myalgia/myositis, sarcoidosis-like conditions, systemic sclerosis-like conditions, Sjögren’s-like/sicca syndrome, lupus-like conditions, and vasculitis [81, 82]. Principally, these irAEs have been observed *de novo* in patients without pre-existing autoimmune diseases. Nonetheless, recent investigations reported that patients with pre-existing autoimmune diseases also suffered rheumatic and systemic irAEs, often manifesting as flares or exacerbations of their known rheumatic disease (in 40% of such patients) or as other types of irAEs (in 35% of such patients) [83–86]. Accordingly, increasing disease activity can typically be managed effectively. Furthermore, the presence of a pre-existing autoimmune disease does not constitute a contraindication and should not prevent the administration of ICIs [81].

### Immunological mechanisms (Integrating T-Cell dysregulation, cytokines, and neutrophils)

ICI-induced inflammatory arthritis presents as inflammatory joint pain, swelling, and stiffness, resembling autoimmune arthritis, such as RA, where the leading mechanism is the dysregulation of immune checkpoints, principally the CTLA-4 and PD-1 pathways, which act as critical regulators of T-cell activation and tolerance [87]. By blocking these regulatory molecules, ICIs promote anti-tumor immune responses through T-cell activation and effector functions; however, this enhanced immune activation can also break immune tolerance, leading to autoimmune manifestations such as arthritis. Evidence suggests that ICI-induced arthritis shares immunopathogenic features with other autoimmune arthritides, including uncontrolled T-cell responses and the production of inflammatory mediators. The genetic deletion of *PD-1* or *PD-L1* induces autoimmunity and organ-specific manifestations [88–90]. Suppressing the PD-1 pathway using ICIs breaks immune tolerance. It enhances T-cell responses, and in the context of ICI-induced arthritis, this heightened T-cell activity can drive autoreactive reactions in the joints. Following activation, autoreactive T cells recruit and infiltrate the synovial tissue, secreting pro-inflammatory cytokines such as IL-17 and TNF-α, which induce hyperinflammation and tissue damage [89, 91, 92].

On the other hand, impaired Tregs, which typically repress autoimmunity, may fail to control autoreactive T-cell activation in the setting of ICIs, leading to arthritis. Additionally, B cells are involved in enhancing the activation and differentiation of autoreactive B cells, thereby promoting the production of autoantibodies. Studies have revealed elevated levels of CXCL13, increased frequency of plasmablasts, and alterations in B cell subsets in patients treated with combined anti-CTLA-4 and anti-PD-1 mAbs [89, 92–95]. In addition to inducing T cells, CTLA-4 inhibition affects monocytes and macrophages, which are responsible for the overexpression of pro-inflammatory cytokines, the enhancement of osteoclastogenesis, and the promotion of bone erosion and joint damage. For instance, PD-1 blockade has been shown to affect M1 macrophages through IL-12, stimulating phagocytosis and releasing pro-inflammatory cytokines, which results in the exacerbation of joint inflammation and damage [96–98]. Monocytes and macrophages respond to the increased T-cell activity by producing pro-inflammatory mediators and differentiating into osteoclasts, which are implicated in bone resorption. The engagement of CTLA-4/Ig fusion proteins with CD80/CD86 on monocytes induces apoptosis. However, this repressive pathway is interrupted during ICI therapy, leading to enhanced monocyte survival and activity [91, 99–101].

Neutrophils are plentiful in the synovial fluid of patients with ICI-inflammatory arthritis and are involved in joint inflammation by releasing TNF-α, proteases, and reactive oxygen species (ROS). The formation of neutrophil extracellular traps (NETs), exposing autoantigens, further activates macrophages, triggers the complement system, and initiates inflammatory autoimmune responses [102–104]. Regarding the crucial role of cytokines in the pathogenesis of ADs, an imbalance between pro-inflammatory mediators such as TNF-α, IL-6, IFN-γ, and anti-inflammatory mediators like IL-10 can provoke inflammation and injury in bone and cartilage. The cytokine milieu in ICI-inflammatory arthritis is characterized by elevated levels of pro-inflammatory cytokines, such as TNF-α, IL-6, IL-17, and IL-23, which drive inflammation in joints by recruiting immune cells, producing autoantibodies, and causing tissue damage and remodeling [105–112].

A cohort study involving patients with ICI-induced arthritis and matched controls revealed significantly elevated serum levels of vascular endothelial growth factor-A (VEGF-A) and TNF-α in the ICI-induced arthritis group, along with increased levels of IFN-γ and IL-6 [113]. These findings indicate that persistent moderate disease activity in 63% of patients occurs beyond six months post-treatment cessation, necessitating extended immunosuppression. Clinically, this highlighted the underreporting in trials, which may underestimate chronic costs and support biomarker-guided therapies for prompt escalation. Mechanistically, ICI inhibition of CTLA-4 and PD-1/PD-L1 precipitated T-cell hyperactivation, promoting synovial lymphoplasmacytic infiltration and cytokine storms (e.g., VEGF-A-induced angiogenesis, TNF-α-driven inflammation), which contrasts with transient irAEs and resembles RA histopathology without autoantibody predominance [113]. An integrated analysis of transcriptomics and chromatin accessibility in human monocytes stimulated with prostaglandin E2 (PGE2) and TNF revealed that PGE2 increased the expression of 41% of TNF-inducible genes, indicating heightened synovial inflammation in RA and ICI-induced arthritis clinically. This synergy modeled 61% of pathogenic macrophage signatures, potentially worsening joint destruction and exacerbations despite NSAID treatment [114]. These increases highlighted the underrecognition of PGE2’s function in maintaining chronicity, as 37% of unique co-induced chromatin peaks were enriched with AP-1 motifs, emphasizing treatment deficiencies in addressing persistent subgroups. PGE2 functioned in conjunction with TNF to modify chromatin through activator protein 1 (AP-1)/CCAAT enhancer-binding protein (CEBP) transcription factors, promoting IL-1β-expressing macrophages and neutrophil chemotaxis. Whereas IFN-γ countered this effect by inhibiting PGE2-enhanced genes via signal transducer and activator of transcription 1 (STAT1)-mediated suppression of AP-1 occupancy and PGE2-induced transcription factors (NR4A1/2, NFE2L2, MAF), establishing a regulatory axis that equilibrates inflammatory responses [113]. This explanation helps to understand the arthritogenic consequences of immunotherapy, suggesting that IFN-γ/PGE2 ratios may serve as indicators for phenotype-specific treatments in ICI-IA [114].

### Clinical features (Polyarthritis vs. PMR-Like patterns)

Severe ICI-induced inflammatory arthritis is characterized by a polyarticular pattern, significant synovial inflammation with neutrophil-dominant effusions, and elevated systemic inflammatory markers, occurring in the absence of infection or prior rheumatic disease. The clinical overlap with culture-negative septic arthritis, characterized by similar synovial fluid profiles, results in diagnostic uncertainty and likely delays in initiating adequate immunosuppressive medication [115]. Despite fluctuations in total synovial white blood cell (WBC) counts among cases, a consistent predominance of neutrophils suggests a common underlying activation of the innate immune system, potentially caused by ICI-mediated dysregulation of tolerance mechanisms within the joint environment, which creates an inflammatory environment. Although similar to infectious arthritis, it lacks microbiological verification, highlighting the importance of including ICI exposure in the differential diagnosis [115].

A recent study demonstrated that malignant tumors considerably suppressed the induction of CIA in mice, signifying that tumors may exert an anti-inflammatory or immunosuppressive effect. However, this inhibition was neutralized when the mice received a combination of anti-PD-1 and anti-CTLA-4 mAbs, exacerbating arthritis; this suggests that while ICIs effectively activate the immune system to battle tumors, they also significantly increase the risk of irAEs, such as arthritis [116, 117]. Remarkably, the combination of ICI therapy not only reversed arthritis suppression but also underscored its dual impact on both cancer and immune response; gene expression analysis revealed a marked upregulation in interferon-alpha/beta receptor subunit 1 (*Ifnar1*) expression in the joints of mice with tumors treated with ICIs compared to controls. Plasma cytokine analysis showed lower IL-6 and higher IFN-γ concentrations, indicating a complex interplay between immune activation and inflammation [116]. These outcomes substantiate the CIA model as a valuable system for studying ICI-induced arthritis and emphasize the necessity of developing treatments that manage arthritis without compromising the efficacy of cancer immunotherapy.

A retrospective analysis comparing ICI-induced arthritis with seronegative and seropositive RA [118]. The study provided substantial clinical insights, revealing that patients with ICI-induced arthritis, predominantly treated with anti-PD-1 therapies, demonstrated a reduced arthritic duration compared to patients with RA. The outcomes indicate a unique temporal profile possibly associated with immune activation resulting from PD-1 pathway interruption; morning stiffness was significantly less common in ICI-induced arthritis compared to RA, and erosive alterations were infrequent, indicating a less destructive phenotype [118]. Mechanistically, ICI-induced arthritis likely results from dysregulated T-cell activation due to checkpoint suppression, resembling the autoantibody profile of seronegative RA (low RF/Anti-CCP positive). Accordingly, ICI-induced arthritis can be a distinct phenomenon, improving comprehension of immunotherapy-induced inflammatory effects and guiding focused treatment approaches.

An observational study evaluating the durability of arthritis induced by ICIs found that active arthritis persisted in more than half of the subjects several months after the cessation of ICIs [119]. A significant percentage continued to exhibit indicators of disease activity in the initial months, underscoring the chronic nature of this disorder. In contrast to self-limiting irAEs, such as colitis, this type of arthritis frequently necessitates prolonged rheumatologic management. The persistence appears to stem from continuous autoimmune activation, even in the absence of ongoing ICI treatment, possibly attributable to enduring T-cell responses after PD-1 or CTLA-4 inhibition. Extended periods of immune checkpoint inhibitor exposure and combination therapy correlated with increased persistence, indicating a more profound alteration of the immune system [119]. The majority of patients necessitated immunomodulatory treatment, which did not compromise cancer management, thus affirming the safety of addressing autoimmune problems in conjunction with oncologic care. A retrospective observational investigation on eight patients with ICI-induced activated osteoarthritis (ICI-aOA) showed symptomatic exacerbation primarily within the first six months after treatment initiation in most cases [120]. However, five instances arose after treatment cessation, suggesting a delayed clinical effect that required extended monitoring and potentially interfered with oncologic care. The data indicated that all patients necessitated therapeutic intervention, with NSAIDs administered to all and intra-articular steroids to 75%, highlighting the significant clinical burden and the insufficiency of conservative therapy for symptom alleviation. Mechanistically, ICI-aOA was associated with PD-1 blockade-induced T-cell hyperactivation, which intensified synovial inflammation through increased production of pro-inflammatory cytokines, such as IL-1β, IL-6, and TNF-α, as well as angiogenesis and macrophage infiltration, thereby worsening the underlying OA phenotypes and distinguishing it from non-inflammatory OA variants [120]. These findings identified ICI-aOA as a unique irAE, enhancing understanding of immunotherapy’s role in revealing dormant inflammatory pathways in OA and promoting increased rheumatologic vigilance in treated patients.

A systematic review of 52 studies identified rheumatic and musculoskeletal irAEs associated with ICIs, revealing a prevalence of arthralgia and myalgia, while arthritis was noted in only five trials and vasculitis in two, indicating potential underreporting and variability that require diligent monitoring to mitigate functional impairment and treatment interruptions [121]. The evidence suggests possible ascertainment bias, as studies frequently highlight high-grade occurrences, hence confounding prevalence estimates and referral decisions. Mechanistically, irAEs were associated with CTLA-4 and PD-1/PD-L1 inhibition, resulting in aberrant T-cell activity and cytokine release (e.g., IL-1β, TNF-α), which induces cross-reactive autoimmunity against synovial antigens, in contrast to temporary non-rheumatic irAEs [121].In addition, examination of 372 cases of ICI-induced arthritis indicated that around 50% manifested polyarthritis, whereas a minority displayed symptoms akin to polymyalgia rheumatica; the majority of patients tested seronegative for RF and anti-CCP [122].

These data underscore the diagnostic intricacy at the oncology-rheumatology interface and reveal a tendency towards documenting severe instances, which may exaggerate treatment discontinuation rates while overlooking milder signs. Arthritis develops due to the lack of immune regulation resulting from CTLA-4 and PD-1/PD-L1 suppression, leading to unchecked T-cell responses, elevation of IL-17 and TNF-α, and infiltration of effector memory CD8^+^ T-cells into the synovium, which differentiates it from autoantibody-mediated RA. The identification of these distinct patterns enhances our comprehension of ICI-related inflammation and facilitates the development of customized therapeutic approaches [122].

### Predictive factors (genetic predisposition and preexisting autoimmune diseases)

Genetic predisposition may stimulate susceptibility to ICI-induced arthritis, with specific HLA alleles and genetic polymorphisms related to ADs, such as SLE and RA, associated with irAE development, including arthritis, in ICI-treated patients [123]. It is probable that these genetic factors shape the dysregulated immune response to ICIs and determine individual susceptibility to ADs [124, 125]. Clinically, ICI-induced arthritis can vary in severity, ranging from mild arthralgia to debilitating polyarthritis. Specific HLA alleles, primarily those associated with RA (e.g., HLA-DRB1*04:05), have been linked to an increased risk of ICI-induced arthritis development, suggesting that some cancer patients may have an intensified risk of autoimmunity following treatment with ICIs [126, 127]. Another systematic review and meta-analysis of 23 trials involving cancer patients with preexisting autoimmune rheumatologic disorders (PADs) treated with ICIs revealed a high prevalence of irAEs; the majority of patients encountered some irAE, with a significant number exhibiting disorders exacerbations or wholly novel autoimmune complications [128]. Patients with RA showed a heightened susceptibility to flares, signifying increased vulnerability within this category. Notwithstanding these limitations, moderate tumor response rates and disease control were observed; however, clinical care frequently required vigilant monitoring and, at times, short treatment pauses [128]. The research highlighted geographical variations, including an elevated incidence of flares in Australian populations, which cautioned that retrospective methods and restricted sample sizes could result in an underestimation of broader risks. Mechanistically, these irAEs were induced by the inhibition of CTLA-4 and PD-1/PD-L1, resulting in hyperactive T-cell responses, cytokine elevations, including IL-1β and TNF-α, and increased autoantibody synthesis [128]. These mechanisms led to brake immune tolerance, which was more evident in patients with preexisting autoimmune disorders compared to those without; the findings endorsed the ongoing application of ICIs in this demographic, emphasizing the necessity of individualized risk evaluation and preemptive inflammatory control approaches (Table 3) [128].

### Diagnosis of ICI-induced arthritis

It has been reported that ultrasonography (US) and magnetic resonance imaging (MRI) have become significant modalities in the diagnosis and monitoring of inflammatory arthritis, as these methods can identify inflammatory characteristics such as synovitis, bursitis, effusions, tendonitis, enthesopathy, tendosynovitis, and structural injuries, including erosions [129]. On the other hand, MRI is considered a potential imaging technique for clarifying muscular involvement in patients with inflammatory myopathies [130]. Positron emission tomography with 2-deoxy-2-[fluorine-18]fluoro-D-glucose, incorporated with computed tomography (18 F-FDG PET/CT), is another well-established imaging technique in oncology; it plays a significant role in evaluating inflammatory states in rheumatology [131]. 18 F-FDG PET/CT is a highly sensitive modality for recognizing both articular and systemic rheumatic conditions and assessing their severity [131]. A study investigated the imaging characteristics of ICI-induced arthritis in a cohort of 19 patients [132]. The researchers identified four clinical patterns of ICI-induced arthritis: RA-like, polymyalgia rheumatica (PMR)-like, psoriatic arthritis (PsA)-like, and oligo-monoarthritis. Most patients were male with a median age of 73 years, and the underlying conditions treated with ICIs included melanoma, lung carcinoma, urothelial cancer, and liver carcinoma [132]. The study utilized various imaging modalities, including US, MRI, and 18 F-FDG PET/CT. The findings showed that RA-like arthritis displayed US findings similar to those of conventional RA, characterized by synovitis and tenosynovitis. PMR-like arthritis frequently involved the hands and wrists, with notable inflammatory changes on US and PET/CT scans. MRI assessments revealed synovitis and bone edema without erosions or myofasciitis [132]. These imaging findings demonstrate that ICI-induced arthritis resembles conventional inflammatory arthropathies, underscoring the importance of careful monitoring and appropriate treatment of these patients.

Another investigation explored employing ultrasound imaging in patients with ICI-induced inflammatory arthritis [133]. The study included nine patients with ICI-induced inflammatory arthritis who underwent musculoskeletal US to assess joint pathology; the outcomes showed that the knees were the most frequently imaged joints, followed by the hands, wrists, feet, and ankles; synovitis was present in 12 of the 18 joints, with an active Doppler signal observed in 50% of these cases [133]. Tendon involvement was frequent, noted in 13 of the 18 joints, presenting as tenosynovitis, tendinitis, and enthesophytes; bone erosions were less common but were observed in three cases, suggesting early structural damage [133]. These results highlight the diverse and significant pathology of ICI-induced inflammatory arthritis, affecting the synovium, tendons, and bones, and emphasize the need for further systematic studies using imaging to improve the management of this novel rheumatic condition.

**Fig. 3:**
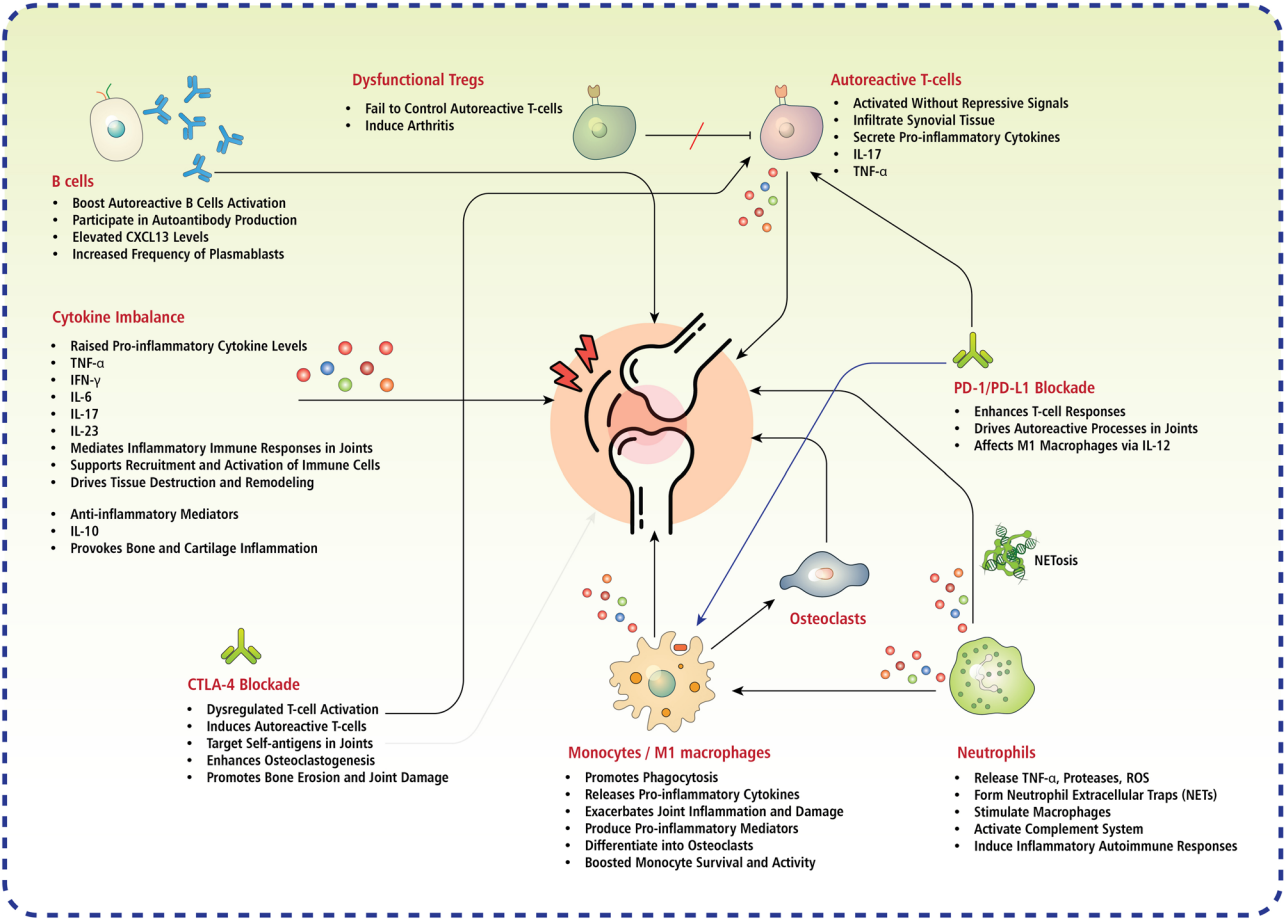
Pathogenic mechanisms in autoimmune arthritis and the impact of immune checkpoint blockade. The mechanisms underlying immune dysregulation and inflammation in autoimmune joint disease concentrate on key subsets of immune cells and imbalances in cytokines. Dysfunctional Tregs cannot adequately suppress autoreactive T cells, thereby permitting their infiltration into synovial tissue and the subsequent release of pro-inflammatory cytokines such as IL-17 and TNF-α, which contribute significantly to joint inflammation and damage. B cells assume a pivotal role by promoting the production of autoantibodies, elevating levels of CXCL13, and enhancing the expansion of plasmablasts. Increased concentrations of inflammatory cytokines, including TNF-α, IFN-γ, IL-6, IL-17, and IL-23, perpetuate chronic inflammation, recruit additional immune cells, and drive the processes of tissue destruction and remodeling. In contrast, anti-inflammatory mediators, such as IL-10, remain insufficient to effectively counteract the inflammatory cascade, resulting in progressive joint damage. Monocytes and M1 macrophages exacerbate inflammation by releasing pro-inflammatory mediators and differentiating into osteoclasts, thereby contributing to bone resorption. Neutrophils exacerbate tissue damage via NETosis, which releases TNF-α, proteases, and ROS while stimulating macrophages and activating the complement system. The implications of ICB, specifically the inhibition of PD-1/PD-L1 and CTLA-4, are elucidated, illustrating their paradoxical role in enhancing T-cell activation and promoting autoimmune responses within the joints by elevating IL-12-mediated M1 macrophage activation. Treg; regulatory T cell, IL-17; interleukin 17, TNF-α; tumor necrosis factor-alpha, B cell; B lymphocyte, CXCL13; C-X-C motif chemokine ligand 13, IL-6; interleukin 6, IL-23; interleukin 23, IFN-γ; interferon-gamma, IL-10; interleukin 10, M1 macrophage; classically activated macrophage, NETosis; neutrophil extracellular trap formation, ROS; reactive oxygen species, PD-1; programmed cell death 1, PD-L1; programmed death-ligand 1, IL-12; interleukin 12, CTLA-4; cytotoxic T-lymphocyte-associated protein 4

Collectively, according to the discussed studies, ICIs have transformed cancer immunotherapy by targeting the CTLA-4 and PD-1/PD-L1 pathways. However, they can also lead to irAEs such as arthritis, which presents as inflammatory polyarticular pain, swelling, and stiffness similar to RA, yet is characterized by a seronegative profile and neutrophil-dominant synovial effusions [118, 122]. ICI-induced arthritis mechanistically results from dysregulated T-cell hyperactivation, wherein the inhibition of regulatory checkpoints permits the activation of autoreactive T cells, leading to pro-inflammatory cytokine surges, including TNF-α, IL-6, IL-17, and VEGF-A, that recruit neutrophils, macrophages, and B cells to synovial tissues, ultimately causing joint damage and osteoclastogenesis in the absence of conventional autoantibodies such as RF or anti-CCP [88, 89, 91, 113, 114]. The interplay between innate and adaptive immunity, exacerbated by dysfunctional Tregs and increased NETosis, resembles autoimmune arthritis but differs in its tumor-related immunosuppressive reversal. This is demonstrated in murine CIA models, where ICI combinations counteract tumor-induced arthritis suppression by upregulating Ifnar1 and modifying the IL-6/IFN-γ ratio [116]. Retrospective assessments indicate that more than 50% of patients experience persistent disease following the termination of ICIs, with polyarthritis being the most common manifestation and erosive alterations being infrequent; this situation requires extended immunosuppression, such as biologics or steroids, without adversely affecting oncologic outcomes [119, 120]. Genetic predispositions, including HLA-DRB1*04:05 alleles, increase vulnerability, especially in individuals with PADs, where exacerbations arise in up to 75% despite significant tumor responses [123, 127, 128]. Systematic reviews highlight underreporting in trials, ascertainment biases that favor severe cases, and the necessity for biomarker-guided therapy (e.g., VEGF-A and TNF-α levels) to alleviate chronicity and functional impairments [121]. The confluence of oncology and rheumatology necessitates diligent monitoring of irAEs, prompt rheumatologic referrals, and tailored methods that balance anti-tumor efficacy with autoimmune concerns to enhance patient outcomes amid the growing indications for ICIs (Fig. 3).

## Is there any relationship between ICI therapy and oral diseases?

Based on the available knowledge, the prevalence of oral irAEs varies according to the type of ICI and the treatment regimen [134]. Combination therapies involving CTLA-4 and PD-1/PD-L1 inhibitors tend to have a higher incidence of irAEs than monotherapy [48, 49]. For instance, in patients treated with anti-CTLA-4 antibodies, higher rates of any grade irAEs and severe irAEs were observed compared to those receiving anti-PD-1/PD-L1 antibodies [50]. ICIs boost immune effector responses by suppressing inhibitory signals that typically preserve immune homeostasis. This can lead to various pathological alterations in the oral cavity, including mucosal inflammation, blister formation, and glandular dysfunction [134].

**Fig. 4:**
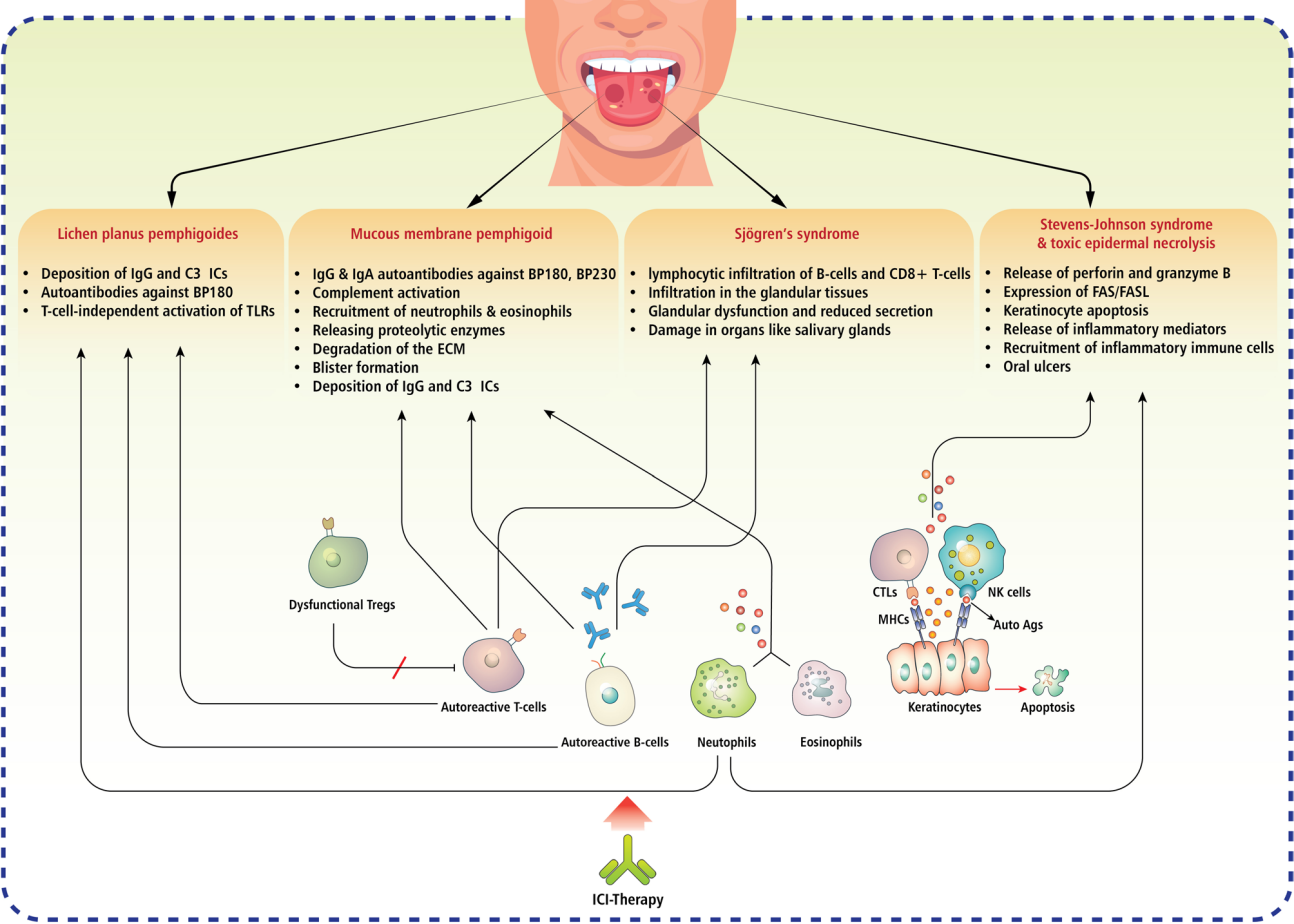
Immune-related mechanisms underlying oral autoimmune disorders associated with ICI therapy. Numerous autoimmune and inflammatory disorders of the oral mucosa may arise as side effects of ICI therapy, including lichen planus pemphigoid, mucous membrane pemphigoid, Sjögren’s syndrome, and Stevens-Johnson syndrome/toxic epidermal necrolysis. Lichen planus pemphigoid is characterized by the deposition of IgG and C3 in the basal membrane zone, with BP180 autoantibodies initiating TLR-mediated, T-cell-independent inflammation and the development of oral lesions. Mucous membrane pemphigoid is mediated by IgG and IgA autoantibodies targeting BP180 and BP230, which activate complement and attract neutrophils and eosinophils that secrete proteolytic enzymes, break down the extracellular matrix, and induce blister formation. Sjögren’s syndrome is characterized by the infiltration of B- and CD8^+^ T-cells into glandular tissue, resulting in diminished salivary production and the promotion of chronic inflammation. Stevens-Johnson syndrome and toxic epidermal necrolysis involve cytotoxic agents, such as perforin and granzyme B, which trigger keratinocyte death through FAS/FASL signaling and result in significant necrosis. ICI therapy intensifies autoimmune processes by impairing immunological tolerance through Treg dysfunction, increased antigen presentation, and the activation of CTL/NK cells, resulting in prolonged inflammation, epithelial injury, and mucosal ulceration. ICI, immune checkpoint inhibitor; IC, immune complex; TLR, toll-like receptor; ECM, extracellular matrix; CTL, cytotoxic T lymphocyte; NK, natural killer; Auto Ag, autoantigen; FAS, Fas cell surface death receptor; FASL, Fas ligand; BP180, bullous pemphigoid antigen 180; BP230, bullous pemphigoid antigen 230; Treg, regulatory T cell

The immune response in oral irAEs often mirrors that of idiopathic autoimmune states but is specifically initiated by ICI therapy. It has been reported that ICIs can be associated with several oral mucosal conditions, including lichen planus-like reactions, bullous pemphigoid, mucous membrane pemphigoid, erythema multiforme, Stevens-Johnson syndrome, and Sjögren-like syndrome. These pathological states can manifest with different clinical features ranging from mild discomfort to severe, life-threatening reactions [134] (Fig. 4).

### Types of oral IrAEs

Lichen planus pemphigoides (LPP) is known as an unusual autoimmune blistering disorder that demonstrates both clinical and histopathologic features of lichen planus (LP) and bullous pemphigoid (BP) or mucous membrane pemphigoid. LPP is categorized as a distinct entity due to autoantibodies against type XVII collagen (COL17, BP180), a critical structural protein in hemidesmosomes at the dermal-epidermal junction. Various medications, including statins, ACE inhibitors, anti-BP180 antibodies, and antituberculosis drugs, have been associated with LPP [135–139].

A study reported a case of a 72-year-old Japanese woman treated with pembrolizumab for primary lung cancer with systemic metastasis, who after six treatment cycles developed pruritic purple-red papules and plaques diagnosed as LP-like dermatitis, progressing to BP characterized by edematous erythemas and tense blisters seven weeks later; histopathology and direct immunofluorescence showed IgG and C3 immune complex deposits at the epidermal basement membrane zone and positive BP180 antibodies [140]. The case demonstrated that LP-like lesions induced by ICIs can precede and potentially prompt BP development via anti-basement membrane zone antibody production, proposing that ICIs may disrupt immune tolerance to the epidermal basement membrane zone; the patient’s condition was exacerbated, and she died due to respiratory complications and carcinoma progression 29 days after BP development, underlining the requirement for awareness and monitoring of such irAEs in patients who received PD-1 inhibitors [140].

Mucous membrane pemphigoid (MMP) is a chronic autoimmune blistering disorder predominantly affecting mucous membranes, including the mouth, eyes, nose, throat, and genitals, characterized by the formation of painful erosions and blisters resulting from autoantibodies targeting components of the basement membrane zone, leading to the separation of the epidermis from the underlying dermis [141, 142]. A study evaluated the case of an 83-year-old woman who developed MMP six months after discontinuing pembrolizumab therapy for metastatic melanoma [143]. Firstly, pembrolizumab led to the complete remission of the melanoma, but the patient later had oral pain with gingivitis and mucosal blistering. The biopsy assessment confirmed MMP, characterized by linear IgG and C3 deposits in the basement membrane zone, though no circulating antibodies were identified. Treatment with doxycycline and topical corticosteroids rapidly controlled and led to a complete remission of MMP, with no relapse detected after a 14-month follow-up, indicating a potential association between pembrolizumab and MMP and highlighting the need for awareness of such irAEs among clinicians [143]. These findings suggest that current drug accountability scoring methods may be inadequate for assessing the prolonged therapeutic effects of immunotherapies.

Stevens-Johnson syndrome (SJS) and toxic epidermal necrolysis (TEN) are severe mucocutaneous reactions marked by widespread epidermal necrosis and detachment. They are classified along a spectrum mainly based on the percentage of the affected body surface area. SJS involves less than 10%, while TEN affects more than 30%, with mucosal involvement being a key feature, including lesions in the oral, ocular, and genital regions [144, 145].

The findings from a case report offered important insights into the irAEs associated with ICIs, where a 55-year-old woman developed SJS/TEN 17 days after her first cycle of pembrolizumab therapy, presenting with a widespread mucocutaneous rash, fever, difficulty breathing, watery eyes, and painful oral ulcers. Physical examination revealed erythematous papules and papulovesicles across her body, including hemorrhagic plaques on the lower lip and buccal mucosa, and a biopsy confirmed the diagnosis of SJS/TEN [146]. This case highlights the importance of recognizing SJS/TEN as a potentially life-threatening irAE associated with ICIs like pembrolizumab, which are increasingly used to treat various cancers. The patient received intravenous acyclovir, methylprednisolone, and supportive care, resulting in the rash resolving within a month. However, her metastatic disease progressed, underscoring the need for clinicians to be vigilant about severe dermatologic reactions in patients on ICI therapy, as these can lead to serious complications such as SJS/TEN and impact treatment decisions and clinical outcomes [146].

Sjögren’s syndrome (SS) is an autoimmune disorder marked by dry mouth and dry eyes caused by the destruction of salivary and lacrimal glands. The pathophysiology involves lymphocytic infiltration, where both CD20^+^ B cells and CD3^+^ T cells, primarily CD8^+^ T cells, invade the glandular tissues, leading to gland dysfunction and decreased secretion. ICIs can also cause irAEs, including Sjögren’s syndrome, by disrupting immune tolerance [147]. The findings from a case report offer valuable insights into irAEs linked to ICIs, where a patient developed SjS-like syndrome during PD-1 inhibitor therapy, exhibiting dry mouth and eyes, with biopsy showing lymphocytic infiltration similar to idiopathic SjS [148]. Another study reported a case of a patient who developed SjS as an irAE following nivolumab treatment for gastric cancer, with symptoms including xerostomia and salivary gland swelling, confirmed by biopsy showing CD8^+^ T-cell infiltration. Treatment involved corticosteroids, leading to symptom improvement, though the cancer progressed [149]. Data from the International ImmunoCancer Registry (ICIR) on sicca/SjS triggered by PD-1/PD-L1 inhibitors revealed that these irAEs often appear suddenly and cause severe dryness. They differ from classic SjS by lower autoantibody positivity and more significant glandular destruction, with treatment usually involving artificial saliva and immunosuppressive therapy [150]. However, cutaneous irAEs are more prevalent than oral manifestations in cancer patients undergoing ICIs, indicating a heightened vulnerability of the skin to immune dysregulation [151]. The simultaneous blockade of PD-1 and CTLA-4 enhances skin toxicity through synergistic T-cell activation, while concurrent chemotherapy exacerbates oral effects due to endothelial and epithelial damage. These toxicities frequently correlate with improved survival, suggesting they may indicate effective immune activation. Nonetheless, retrospective analyses are constrained by inconsistent follow-up and underreporting of mild or subclinical events, which impede a precise understanding of resistance mechanisms [151].

Converging evidence from the reviewed studies indicates that oral irAEs manifest as various blistering, erosive, or glandular disorders, with a higher incidence in combination therapies. However, different clinical outcomes highlight variability in severity, ranging from mild lichenoid reactions to life-threatening SJS/TEN, emphasizing the importance of early recognition to prevent complications such as respiratory failure or carcinoma progression (Table 4).

**Table 4:** Summary of oral IrAEs following treatment with ICIs

Intervention/condition	Study type & population	Mechanism/pathophysiology	Key results & quantitative findings	Ref.
**Pembrolizumab-induced LP-like lesion preceding Bullous Pemphigoid (BP)**	Case report; 72-year-old woman with metastatic lung cancer, 6 cycles pembrolizumab	PD-1 blockade → autoantibody production against BMZ (BP180) → transition from LP-like dermatitis to BP	• LP-like lesion with IgG deposition at BMZ before BP onset; BP180 level: 115.7 U/mL; improvement with prednisolone, death due to cancer progression 29 days after BP onset	[140]
**Pembrolizumab-associated Mucous Membrane Pemphigoid (MMP)**	Case report; 83-year-old woman, metastatic melanoma, MMP onset 6 months post-pembrolizumab	PD-1 blockade → autoimmune blistering of mucosal BMZ (IgG, C3 deposits)	• Rapid remission with doxycycline + topical corticosteroids; no relapse after 14 months; no circulating autoantibodies detected	[143]
**Pembrolizumab-induced SJS/TEN**	Case report; 55-year-old woman, metastatic cancer, onset 17 days after 1 st dose	Severe T-cell–mediated cytotoxicity against keratinocytes	• Mucocutaneous rash, mucosal erosions; confirmed SJS/TEN; recovery after methylprednisolone + acyclovir + supportive care; disease progressed	[146]
**ICI-associated Sicca/Sjögren Syndrome**	Multicenter registry study (ICIR); 26 patients, mean age 63.6y, 42% female; cancers: lung (46%), renal (27%), melanoma (15%)	PD-1/PD-L1 blockade → immune-mediated exocrinopathy with organ-specific autoimmunity	• Dry mouth 96%, dry eye 65%; biopsy: chronic/focal lymphocytic sialadenitis (focus score mean 1.8); ANA 52%, anti-Ro 20%; systemic features in 89%; systemic therapy response: 73%	[150]
**Oral toxicities in ICI trials**	Meta-analysis; 95 clinical trials	Class effect of ICIs on oral mucosa and salivary glands	• Xerostomia 5%, mucositis/stomatitis 3%, dysgeusia 3%, oral pain 3%, dysphagia 2%, candidiasis 2%; rare/absent lichenoid or pemphigoid reactions	[6]
**Oral irAEs during ICI therapy**	Single-center observational study; 165 patients	PD-1/PD-L1 or combination blockade ± chemotherapy → oral mucosal inflammation	• 38.2% developed oral mucositis/xerostomia; grade 3 mucositis in 4 patients (discontinuation in 3); oral care improved grade 1–2 cases	[239]
**Cutaneous & Oral irAEs prevalence & OS impact**	Retrospective study; 748 cancer patients on ICIs	Immune dysregulation with PD-1 + CTLA-4 or ICI + chemotherapy	• Cutaneous irAEs: 55%; oral lesions: 11%; xerostomia: 9%; median onset: 11w (cutaneous/oral), 21.5w (xerostomia); oral + cutaneous irAEs associated with better OS (*p* = 0.0001)	[151]

### Shared mechanisms (autoantibody production and T-Cell infiltration)

MMP is caused by an autoimmune response where IgG and IgA autoantibodies target adhesion molecules at the dermo-epidermal junction [143]. Key targeted components include BP180 (type XVII collagen) and BP230, which are essential for maintaining skin integrity and mucosal barriers. This binding activates the complement system, leading to an inflammatory cascade. Inflammatory cells, such as neutrophils and eosinophils, are recruited to the site and release proteolytic enzymes that degrade the extracellular matrix, resulting in blister formation [143]. The PD-1/PD-L1 pathway is essential for maintaining immune tolerance, and pembrolizumab, by blocking PD-1, disrupts this tolerance, potentially leading to autoimmunity; losing regulatory control over T-cells can heighten autoreactive T-cell responses, contributing to MMP pathology [148]– [149]. It has been proposed that blocking the PD-1/PD-L1 pathway with ICIs increases the production of antibodies against the hemidesmosomal protein BP180; an imbalance among autoreactive Th cells, Tregs, and T-cell-independent activation of TLRs may trigger the release of autoantibodies, with tumor expression of the BP180 antigen supporting the production of anti-BP180 antibodies [152–154]. SJS and TEN involve a complex interaction of immune system components that drive their development, with CTLs and NK cells playing a central role. These cells become abnormally activated in response to drug antigens presented by MHC molecules on the surface of keratinocytes. They release cytotoxic molecules such as perforin and granzyme B, which cause keratinocyte apoptosis. Additionally, granulysin, produced by these cytotoxic cells, acts as a potent mediator of cell death in SJS/TEN and is elevated in the serum during the early stages [155]. Additionally, the Fas-FasL interaction promotes keratinocyte apoptosis, as Fas ligand (FasL) on T-cells binds to the Fas receptor on keratinocytes, triggering cell death. This immune-mediated damage is further exacerbated by inflammatory cytokines and chemokines, which enhance the recruitment and activation of additional immune cells, thereby creating a cycle of ongoing damage [155].

The implications are significant in the context of ICIs that block inhibitory molecules, which usually keep the immune system in check. While this boosts the body’s ability to fight cancer, it also increases the risk of irAEs, including severe skin reactions such as SJS and TEN, as the increased T-cell activity caused by ICIs inadvertently attacks skin cells, resulting in severe symptoms [156–158]. The pathophysiology involves the release of inflammatory cytokines and chemokines, which further amplify the immune response and cause widespread tissue damage [158]. This hyperactivation of the immune system highlights the delicate balance needed when managing cancer patients with ICIs, requiring careful monitoring for early signs of adverse reactions and quick intervention to prevent severe outcomes [158]. ICI therapy may strengthen autoimmune reactions by disrupting immune tolerance mechanisms. Dysfunctional Tregs fail to suppress autoreactive T and B cells, leading to increased inflammation and tissue damage. Additionally, ICI therapy may enhance antigen presentation and activate CTLs and NK cells, potentially further damaging keratinocytes and epithelial structures in the oral mucosa [134, 152–160].

Converging mechanistic evidence from the reviewed investigations highlights the role of autoantibody production (e.g., anti-BP180 IgG/IgA) and T-cell infiltration (CD8^+^ CTLs, NK cells) in causing blistering and mucosal erosion, often enhanced by complement activation and cytokine release. However, different contexts demonstrate that PD-1 blockade more strongly disrupts tolerance in glandular tissues (SjS-like) compared to CTLA-4 inhibition, which increases cytotoxic responses in severe reactions, such as SJS/TEN.

## Association between oral disorders and arthritis

### Clinical and epidemiological evidence

**Fig. 5:**
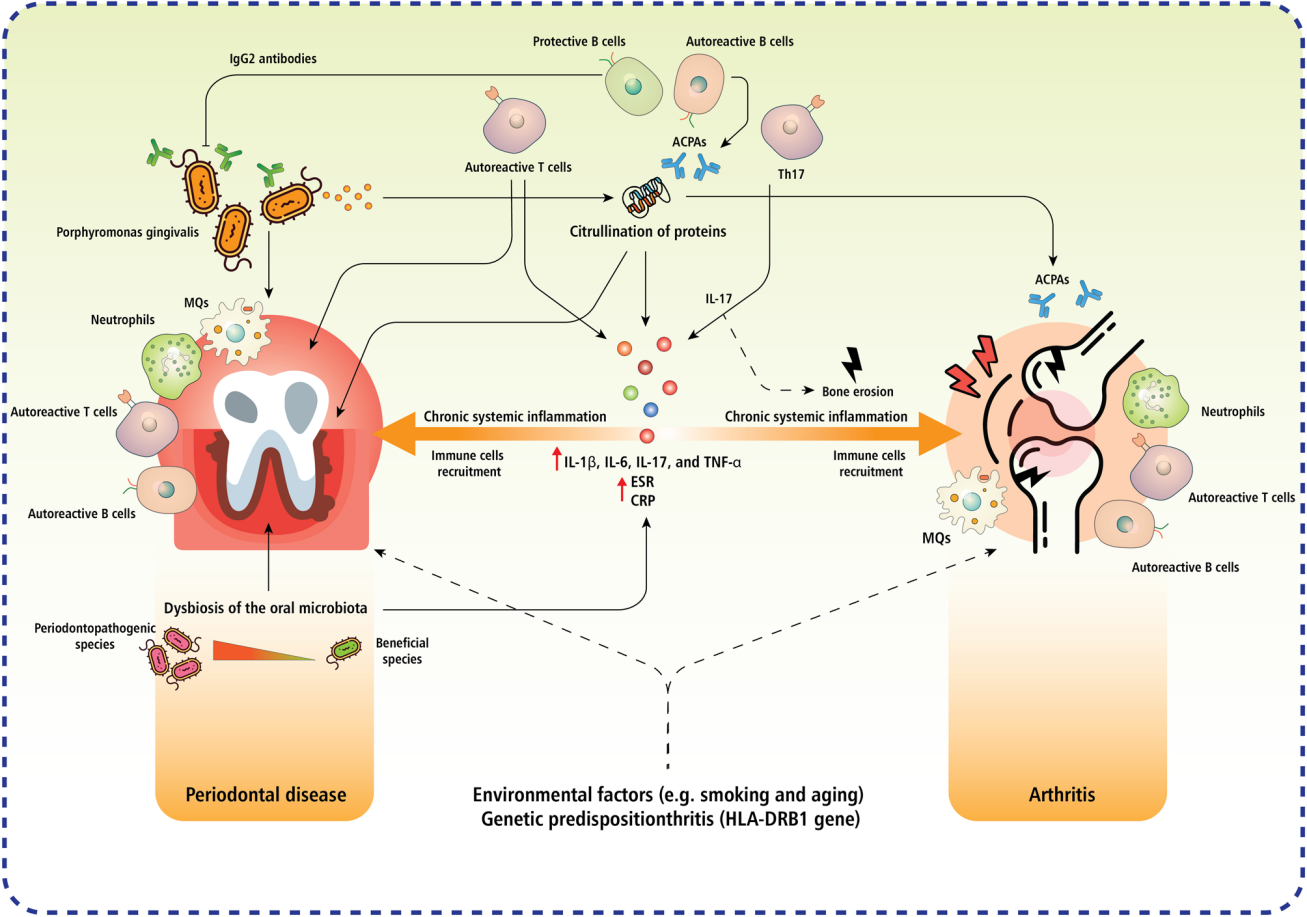
Periodontal disease is a significant contributor to systemic inflammation and the development of arthritis. The connection between PD and arthritis highlights chronic systemic inflammation as a common pathogenic mechanism. Dysbiosis of the oral microbiota, particularly the overgrowth of *P. gingivalis*, triggers local immune responses that result in the recruitment of neutrophils and macrophages, the production of IgG2 antibodies, and the activation of autoreactive B and T cells. Chronic periodontal inflammation induces the systemic release of cytokines, including IL-1β, IL-6, IL-17, and TNF-α, which contribute to the production of ACPAs and the activation of Th17 cells. These immune-mediated processes promote joint inflammation, cartilage degradation, and bone erosion, establishing a mechanistic linkage between PD and arthritis. Environmental factors such as tobacco use and aging, along with genetic predisposition (specifically HLA-DRB1), further enhance susceptibility to ACPA production and immune dysregulation. Elevated levels of inflammatory markers, including ESR and CRP, indicate systemic inflammation and disease progression. The bidirectional relationship between PD and arthritis underscores the impact of chronic oral inflammation on systemic autoimmunity. MQ, macrophage; IL, interleukin; TNF-α, tumor necrosis factor-alpha; ESR, erythrocyte sedimentation rate; CRP, C-reactive protein; HLA, human leukocyte antigen; ACPA, anti-citrullinated protein antibody; Th17, T helper 17; *P. gingivalis*, *Porphyromonas gingivalis*

Recent years have observed a growing acknowledgment of the relationship between oral disorders, especially periodontitis, and RA [161]. Clinical and epidemiological studies consistently indicate that people with RA have a greater prevalence and severity of periodontitis compared to healthy individuals [162–164]. Severe periodontitis is associated with increased RA disease activity and severity, indicating a bidirectional relationship between these pathologic states. Both conditions are characterized by persistent immune-mediated inflammation. Their simultaneous occurrence is significantly greater than what would be expected by chance [165] (Fig. 5).

Individuals at risk for pre-RA. Those with early RA exhibit heightened periodontal indices (plaque index, bleeding on probing, probing depth) as well as elevated systemic biomarkers such as anti-citrullinated protein antibodies (ACPAs) and erythrocyte sedimentation rate (ESR), suggesting that periodontal inflammation may precede or exacerbate systemic autoimmunity [166]. In established RA cohorts, periodontal disease is nearly uniformly observed, frequently in advanced stages [167]. Meta-analyses demonstrate significantly increased clinical attachment loss, probing depth, and bleeding on probing (BOP) in RA patients relative to controls [168]. These studies collectively indicate that periodontal disease is a clinically significant factor that affects the onset, activity, and course of RA, rather than merely an incidental comorbidity.

### Common immunopathogenic pathways

RA and periodontal disease exhibit several immunopathogenic pathways that culminate in persistent inflammation and tissue damage. Pro-inflammatory cytokines, including IL-1β, IL-6, IL-17, and TNF-α, serve as pivotal mediators in both disorders [169]. Th17-derived IL-17 promotes osteoclastogenesis and bone erosion in both synovial and periodontal tissues, while increased cytokine levels are observed in the blood, synovial fluid, and gingival crevicular fluid of RA patients. Aberrant activation of T and B lymphocytes constitutes another unifying characteristic. In RA, autoreactive T cells and B cells contribute to the generation of ACPAs, a characteristic that is indicative of disease severity [170]. Likewise, periodontal disease is correlated with B-cell hyperactivity and elevated levels of B-cell-activating factor (BAFF) and a proliferation-inducing ligand (APRIL), which stimulate autoantibody production and sustain inflammation [171]. The upregulation of MMPs in both scenarios facilitates the breakdown of connective tissue. Moreover, elevated salivary IFN-α2 in periodontal disease reflects the interferon profile observed in RA, suggesting that innate antiviral mechanisms may contribute to persistent immune activation [167]. These convergent pathways underscore a common inflammatory network that underlies both joint and periodontal disease.

### Microbial dysbiosis, genetic susceptibility, and environmental catalysts

Oral microbial dysbiosis is a crucial characteristic connecting RA and PERIODONTAL DISEASE. High-throughput sequencing uncovers unique oral microbiome profiles in RA patients, characterized by an abundance of periodontopathogenic species [172]. *Porphyromonas gingivalis* is notably significant for its capacity to express peptidylarginine deiminase (PAD), which facilitates the citrullination of proteins [173, 174]. Citrullinated antigens induce the development of ACPAs, which are crucial in the pathophysiology of RA [170, 175]. The bacterial load of *P. gingivalis* is associated with periodontal attachment loss in RA-periodontal disease patients, highlighting its role in linking local microbial damage to systemic autoimmune disease [176].

These microbial elements engage with host vulnerability. Common genetic risk alleles, including *HLA-DRB1* polymorphisms, enhance susceptibility to both RA and periodontal disease via influencing antigen presentation and immunological responses [177]. Environmental exposures, particularly smoking and age, significantly increase risk [178, 179]. Mendelian randomization studies enhance causal inference by demonstrating that RA predisposes individuals explicitly to chronic, rather than acute, periodontal disease [180, 181]. This differentiation reinforces the perspective that prolonged autoimmune dysregulation, via persistent osteoclastogenesis, matrix degradation, and chronic cytokine secretion, creates a conducive environment for periodontal inflammation that extends beyond temporary microbial infection [182, 183].

### Systemic inflammatory interactions among autoimmune disorders

As discussed, local periodontal inflammation may stimulate systemic immunological activation, hence contributing to the persistence and advancement of RA. In both cases, increased ESR and C-reactive protein (CRP) levels indicate this reciprocal amplification [184]. The release of inflammatory mediators from periodontal tissues, such as TNF superfamily members, interferons, and MMPs, can intensify synovial inflammation, resulting in a self-sustaining inflammatory cycle [171, 185].

The link between RA and periodontal disease is not singular. Meta-analytic research suggests that people with SLE may exhibit equivalent or heightened vulnerability to periodontal disease compared to those with RA [186]. SLE-specific processes, such as immune complex deposition, complement activation, and IFN-α dysregulation, appear to interact with periodontal inflammation, thereby accelerating tissue destruction. This observation contests the RA-centric viewpoint and underscores the necessity to broaden the study and monitoring of periodontal disease across autoimmune disorders. Notably, not all forms of arthritis exhibit identical patterns: research indicates no substantial disparity in periodontal disease incidence between RA and OA, despite elevated bleeding on probing in OA [168]. This suggests that RA-specific autoimmune mechanisms, particularly abnormal citrullination and the production of ACPAs, are crucial to the RA-periodontal disease axis, distinguishing it from non-autoimmune inflammatory conditions (Table 5).

**Table 5:** Association of periodontal disorders with arthritis

Study type	Study details	Mechanisms	Key results (with important figures)	Ref
Cross-Sectional Study	Evaluated periodontal condition, *P. gingivalis* presence, and IgG subclasses in Pre-RA (*n* = 119), eRA (*n* = 36), and matched controls (*n* = 155). Full-mouth exams measured PI, BOP, PD, CAL; ELISA for IgG; PCR for P. gingivalis. Blinded periodontist; matched for age, sex, smoking.	• Autoantibodies (RF, ACPA) precede RA • *P. gingivalis* citrullination links PD to RA via immune response • IgG subclasses indicate activity • Inflammation markers (hsCRP, ESR) correlate with PD severity.	• Pre-RA had higher PD indices (PI *p* = 0.001, BOP *p* = 0.003, PD *p* = 0.034, CAL p = NS) and periodontitis prevalence (92% vs. 70%, *p* = 0.002) than controls • eRA showed no significant PD difference but higher IgG1/IgG2 (*p* = 0.001) • *P. gingivalis* more prevalent in controls (*p* = 0.001); ACPA associated with severe PD (*p* = 0.031).	[166]
Cross-Sectional Study	Assessed salivary/serum inflammatory mediators (e.g., IL-6, TNF-α, MMP-8/9) in RA patients (*n* = 70) with varying PD stages (II: *n* = 22; III/IV: *n* = 48). Saliva/serum collection; multiplex assays; periodontal exams (PPD, CAL, BOP); logistic regression for associations.	• Inflammatory mediators (cytokines, MMPs) drive PD progression in RA • TWEAK/TNFSF12 correlates with bleeding pockets • Shared pathways of inflammation and tissue destruction via TNF receptors and MMPs.	• Higher salivary TWEAK (OR = 1.001, *p* = 0.024) and sTNF-R2 (OR = 1.002, *p* = 0.020) in stage III/IV PD; serum IL-6 higher in stage III/IV (*p* = 0.025) • Correlations: salivary TWEAK with BOP (*r* = 0.45, *p* < 0.01) • No sex differences in mediators.	[167]
Mendelian Randomization Study	Two-sample MR using GWAS data (RA: *n* = 58,284; chronic PD: *n* = 17,353; acute PD: *n* = 4,486); 16 SNPs as IVs for RA; IVW, MR-Egger, weighted median/mode for causality; sensitivity analyses (funnel plots, leave-one-out).	• Genetic variants (e.g., HLA-DR4) link RA to PD via citrullination and immune dysregulation • Pleiotropy tested; RA as risk factor for chronic PD but not acute.	• RA causally associated with chronic PD (OR = 1.03, 95% CI: 1.01–1.05, *p* = 0.002) but not acute PD (OR = 1.00, *p* = 0.98) • No pleiotropy (MR-Egger intercept *p* = 0.85); heterogeneity low (I²=0%).	[181]
Cross-Sectional Study	Measured salivary HLA-DR4, MMP-8, ACPA, and P. gingivalis load in PD + RA (*n* = 30), PD (*n* = 30), and controls (*n* = 20); ELISA for biomarkers; qRT-PCR for bacterial load; DAS-28 for RA activity; ROC for diagnostic accuracy.	• HLA-DR4 susceptibility allele in RA-PD link • MMP-8 in tissue destruction; ACPA via citrullination by *P. gingivalis* • Bacterial load exacerbates inflammation.	• Lower HLA-DR4 in PD groups (*p* < 0.05) • Higher MMP-8/ACPA in PD + RA (MMP-8: 45.2 pg/ml vs. 20.1 in controls, *p* < 0.001) • *P. gingivalis* higher in PD + RA (load: 5.6 log copies vs. 3.2, *p* < 0.01) • ROC AUC for MMP-8 = 0.85; correlations: MMP-8 with CAL (*r* = 0.62, *p* < 0.01).	[176]
Systematic Review and Meta-Analysis	Reviewed 17 studies (RA: *n* = 49,318; SLE: *n* = 7,400); pooled prevalence of PD; subgroup by PD definition, region, disease duration; random-effects model; heterogeneity (I²).	• Shared autoimmune pathways (e.g., cytokines like TNF-α, IL-6) • Immunosuppression increases PD risk • Microbial dysbiosis in oral-gut axis.	• PD prevalence: 57% in RA (95% CI: 46–67%, I²=98%), 35% in SLE (95% CI: 22–50%, I²=97%) • Higher in Asia (RA: 65%, *p* < 0.01); no difference by disease duration (*p* = 0.45).	[186]
Systematic Review and Meta-Analysis	Included 17 studies (RA: *n* = 1,533; controls: *n* = 1,305); meta-analysis of PD risk, clinical parameters (CAL, PPD); Downs & Black appraisal; random-effects; publication bias (Egger’s test).	• Autoantibodies (ACPA, RF) and citrullination by *P. gingivalis* link RA-PD • Inflammation via cytokines (TNF-α, IL-1); bone erosion shared.	• Higher PD risk in RA (RR = 1.13, 95% CI: 1.04–1.23, *p* = 0.006, I²=89%) • Greater CAL (MD = 1.1 mm, *p* < 0.001), PPD (MD = 0.9 mm, *p* = 0.002) • Bias low-moderate (scores 15–22/27).	[168]

Taken together, the intricate relationship among periodontal disease, RA, and systemic autoimmunity has significant clinical ramifications. Periodontal disease in RA should be considered not as an accidental comorbidity, but as a fundamental aspect of the systemic inflammatory load. Timely recognition and collaborative care of periodontal disease may mitigate systemic inflammation, regulate autoimmunity, and possibly modify the progression of RA [166, 187]. Future longitudinal and interventional studies are needed to determine if targeted periodontal therapy can reduce systemic inflammatory burden, improve rheumatologic outcomes, and serve as an adjunctive approach in the management of autoimmune diseases. Integrating periodontal health into conventional rheumatology care may signify a paradigm change, expanding the reach of precision therapy in chronic autoimmune diseases.

## Management strategies for ICI-related rheumatological and oral adverse events

ICIs have significantly transformed cancer therapy by enhancing the anti-tumor immune response. Nevertheless, their administration has been linked to a range of irAEs, including rheumatic manifestations such as arthritis and oral disorders. Research has shown that managing ICI-induced arthritis requires a comprehensive strategy that effectively balances the regulation of inflammation with the preservation of the ICI-mediated anti-tumor effect.

### Clinical presentation and initial management

ICI-induced arthritis can manifest in various characteristics, including symmetrical RA-like seronegative arthritis, seropositive RA, and asymmetrical arthritis, sometimes accompanied by psoriasis or arthralgia [188]. The preliminary management often involves employing NSAIDs, systemic glucocorticoids, conventional synthetic disease-modifying antirheumatic drugs (csDMARDs), and biologic disease-modifying antirheumatic drugs (bDMARDs) [189]. The European League Against Rheumatism (EULAR) recommends treating ICI-induced irAEs first with NSAIDs and/or analgesics for mild-to-moderate rheumatic irAEs [190, 191]. However, regarding the low efficiency (below 60%) of NSAIDs, monotherapy with these drugs needs to be improved, and using more potent immunomodulatory treatments to manage inflammation is necessary. Accordingly, NSAIDs are usually used to prevent the immunosuppressive impacts of glucocorticoids, which might interfere with the anti-tumor response of ICIs [192].

### Glucocorticoid therapy

Glucocorticoids are regarded as potential therapeutic agents in managing ICI-induced arthritis. Approximately two-thirds of patients are administered systemic glucocorticoids, with initial dosages ranging from 10 to 20 mg of prednisone equivalent per day, which are subsequently adjusted according to the severity of the arthritis [193, 194]. Higher doses, up to 40 mg/day, may be used in severe cases [81]. Generally, patients respond well to glucocorticoid therapy, with symptom control achieved in almost all cases [195]. Intra-articular glucocorticoid injections are also administered in about 15% of patients, principally those with mono- or oligoarthritis, resulting in favorable local responses [81]. The duration of glucocorticoid tapering differs from a few weeks to several months and is often prolonged if symptoms recur. Some patients remain on low to moderate doses to maintain ICI therapy while managing irAE symptoms. Notably, no severe adverse effects from glucocorticoids have been reported, although caution is advised regarding potential interference with tumor response [119].

### CsDMARD and bDMARD therapy

In cases where patients exhibit insufficient responsiveness to glucocorticoids or necessitate glucocorticoid-sparing treatment protocols, csDMARDs and bDMARDs are considered suitable alternatives. Approximately 20% of patients with ICI-induced arthritis are treated with csDMARDs, with MTX being the most common choice (60%), followed by hydroxychloroquine and sulfasalazine, either alone or in combination [189]. csDMARDs enable glucocorticoid tapering and symptom control in about 90% of cases, with complete arthritis control reported in several studies [196]. In this context, a study explored the characteristics and treatment of new-onset arthritis induced by ICIs [197]. The findings revealed that ICI-induced arthritis predominantly affects males and presents various patterns, such as monoarthritis, oligoarthritis, and polyarthritis. The pathologic state is not self-limiting, with disease activity frequently increasing upon prednisolone tapering. Initial treatment with MTX demonstrated promising efficacy and safety, especially in cases of polyarthritis. Imaging techniques, such as PET-CT, were effective in detecting ICI-induced arthritis. Overall, patients with ICI-induced arthritis also tend to exhibit a better tumor response to the therapy [197]. The findings underscore the importance of early and appropriate intervention in the effective management of arthritis, with the objectives of achieving remission and improving the quality of life while preserving the integrity of cancer treatment. Biologic DMARDs are utilized in approximately 10% of patients. The primary biologics administered include TNF inhibitors, which account for 70%, and tocilizumab, which comprises 30%. This combination demonstrates notable efficacy in alleviating symptoms of arthritis. Tocilizumab, which targets the IL-6 receptor, is particularly effective in patients who exhibit inadequate responses to TNF inhibitors [198].

### Optimal therapeutic strategies for ici-induced arthritis: protocols and effectiveness

Due to the lack of extensive randomized controlled trials, existing therapeutic protocols predominantly depend on expert consensus, retrospective cohort studies, and case series [199, 200]. Furthermore, varying degrees of severity, categorized as mild, moderate, and severe, will influence the aggressiveness of treatment approaches.

Patients diagnosed with mild arthritis (grade 1) typically report minimal or absent joint pain and stiffness, with no significant impairment in functionality. The primary management strategy involves the administration of NSAIDs and intra-articular corticosteroid injections, aimed at alleviating localized symptoms while minimizing systemic immunosuppression [201]. Although NSAIDs have demonstrated efficacy in addressing mild inflammation, their usage is somewhat limited due to associated risks of gastrointestinal, renal, and cardiovascular complications, particularly among cancer patients [199, 202]. In moderate arthritis (grade 2), evident joint involvement and functional limitations necessitate the incorporation of systemic glucocorticoids. Prednisone or its equivalent, prescribed at a dosage of 10–20 mg/day, is generally recommended, with a gradual tapering schedule over several weeks or months contingent upon the clinical response [203]. When symptoms fail to improve or deteriorate despite tapering, initiating DMARDs such as MTX, leflunomide, or sulfasalazine is warranted [87]. MTX, administered at a dosage of 10–20 mg/week alongside folic acid supplementation, is regarded as effective for reducing inflammation and preventing joint damage, exerting a limited impact on ICI efficacy in most patients [204].

Grade 3 to 4 severe cases with polyarthritis, significant functional impairment, or glucocorticoid dependence require a more aggressive approach. Initial treatment can involve high-dose glucocorticoids (0.5–1 mg/kg/day of prednisone equivalent), which should be tapered quickly to minimize the duration of immunosuppression [87]. TNF inhibitors are effective for steroid-refractory arthritis, while IL-6 inhibitors may be preferred in cases with systemic inflammatory features [24]. Small-molecule JAK inhibitors (tofacitinib) are being developed as alternative options, but their impact on ICI efficacy has yet to be established [205].

Research demonstrates that administering rheumatic diseases with DMARDs for rheumatic diseases at the initial phases enhances the control of clinical features [206]. Additionally, it reduces reliance on glucocorticoid therapy, thereby mitigating the risk of tumor progression associated with immune suppression, surpassing the threshold required to alleviate clinical symptoms. Retrospective analyses reveal that MTX and TNF inhibitors do not significantly compromise the efficacy of ICIs [207]. However, prolonged high-dose glucocorticoid treatment (exceeding 10 mg/day prednisone equivalent) appears to correlate with poorer survival outcomes in certain malignancies [208]. Given these considerations, a multidisciplinary strategy involving oncologists and rheumatologists is essential for tailoring therapy according to disease severity and individual patient characteristics.

### Discontinuation and reintroduction of ICI therapy

ICI therapy is discontinued in approximately one-quarter of patients due to ICI-induced arthritis [81]. However, most patients can resume ICI therapy once arthritis is controlled, provided the adverse event is not life-threatening (grade 4) [209]. Deciding to discontinue or reintroduce ICIs involves carefully assessing the severity of irAEs, the need for immunosuppression, the cancer stage, and engaging in a shared decision-making process with the patient. The management of ICI-induced arthritis requires a nuanced approach that includes NSAIDs, glucocorticoids, csDMARDs, and bDMARDs, tailored to the severity of arthritis and the patient’s response to treatment. Glucocorticoids are effective for most patients; however, csDMARDs or bDMARDs should be considered for those with insufficient responses or who require glucocorticoid sparing. The ultimate goal is to control arthritis symptoms while allowing the continuation of effective ICI therapy for cancer treatment. Continuous clinical monitoring and collaboration between rheumatologists and oncologists are essential to optimize outcomes and balance the control of rheumatic irAEs with anti-tumor efficacy [188].

Collectively, achieving a balance in the administration of therapies is essential for the effective management of arthritis, as maintaining anti-tumor immune responses presents a persistent clinical challenge. Ongoing investigations aim to identify predictive biomarkers associated with ICI-induced arthritis and to develop targeted treatments that mitigate autoimmune toxicities while preserving anti-tumor immune efficacy [210]. Furthermore, approaches to improve immune tolerance and restore immune homeostasis are being studied to inhibit or diminish irAEs while amplifying ICI-mediated therapeutic advantages in patients with malignancies [211].

### Management of ICI-induced oral disorders

ICI-induced oral disorders result from nonspecific immune activation, impacting around 6.8–10% of patients during the initial year of treatment, according to research on PD-1/PD-L1 inhibitors [13, 212]. The clinical rationale emphasizes the importance of early recognition and intervention to alleviate symptoms and avert severe complications, including mucosal ulceration or systemic infections, particularly in patients undergoing immunosuppressive therapies such as steroids [213]. Regular monitoring and patient education are crucial for promptly identifying issues, ensuring effective cancer treatment, and maintaining oral health.

Oral mucositis occurs in up to 2% of patients receiving nivolumab or pembrolizumab for head-and-neck squamous cell carcinoma. Treatment options include topical corticosteroids to alleviate inflammation, analgesics for pain management, and topical antifungals to avert secondary infections [15]. Severe cases may necessitate the use of transdermal fentanyl for pain management, in conjunction with antimicrobial doses of doxycycline (100 mg/day) and corticosteroids. Additionally, photobiomodulation employing low-level lasers (e.g., 670–830 nm GaAlAs, 660 nm InGaAlP, or 940 nm diode lasers) has demonstrated efficacy [15]. Xerostomia, which develops 2–8 months after ICI therapy, is treated with topical oral lubricants, saliva stimulants (such as pilocarpine 5 mg or cevimeline 30 mg, administered three times daily for 3 months), saliva substitutes, and oral lozenges containing anhydrous crystalline maltose. Additional interventions include topical physostigmine, amifostine, electrostimulation, and photobiomodulation to mitigate symptoms and decrease dental complications such as caries [15]. Dysgeusia occurs in fewer than 3% of patients receiving PD-1/PD-L1 inhibitors. It can be managed with oral zinc supplements (such as zinc gluconate), amifostine, and low-level laser therapy applied to the dorsum of the tongue [15]. Lichenoid reactions may arise months after initiating ICI and are typically managed with topical corticosteroids. Systemic corticosteroids are reserved for more extensive cases, with approximately 25% of these reactions being dermal and lacking mucosal involvement [15]. In cases of severe or combined lesions, including Stevens–Johnson syndrome or bullous pemphigoid, modifications or cessation of ICI therapy may be necessary for Grades 3 and 4 toxicities, necessitating specialist consultation for complex case management [15].

Effective preoperative oral health management is essential for patients receiving ICI before surgery, as inadequate oral health may elevate the risk of postoperative complications. A study involving 70 oral cancer patients from 2008 to 2014 found that preoperative oral care significantly decreased postoperative inflammation, including professional teeth cleaning and scaling within three days before surgery. The oral care group exhibited lower CRP levels on day 1 (5.69 mg/dL vs. 7.90 mg/dL, *P* = 0.030) and on days 3 to 5 (6.15 mg/dL vs. 6.97 mg/dL) compared to the non-oral care group for severely invasive surgeries [214]. These findings suggest that preoperative oral care may benefit patients experiencing oral irAEs or those undergoing steroid treatment, potentially mitigating inflammation and the risk of infection during surgical interventions.

Patients receiving ICI treatment, especially those administered systemic steroids for irAEs, exhibit heightened risks for infections, including opportunistic infections (OIs). A retrospective study involving 112 patients treated with ICIs and steroids (≥ 20 mg prednisone equivalent for ≥ 4 weeks) indicated an infection rate of 20%, with 7% classified as opportunistic infections, including oral candidiasis (*n* = 4) and *Pneumocystis jirovecii* pneumonia (PJP) (*n* = 1) [215]. The National Comprehensive Cancer Network (NCCN) guidelines advocate for PJP prophylaxis using sulfamethoxazole/trimethoprim in patients undergoing prolonged steroid treatment. However, 43% of individuals who received this prophylaxis subsequently developed infections, prompting questions regarding its effectiveness [215]. Doxycycline (100 mg/day) is utilized to manage ICI-induced oral mucositis, primarily for treatment rather than prophylaxis [15]. Patients undergoing oral surgery may benefit from general preoperative antibiotic prophylaxis principles, including administering cefazolin 30–60 min before incision to target pathogens such as *Staphylococcus aureus*. This is especially relevant for immunocompromised patients or those experiencing severe oral irAEs [216]. Specific guidelines for antibiotic prophylaxis related to oral health in patients treated with ICIs are limited, indicating a need for further research (Table 6).

**Table 6:** Management strategies for ICI-related rheumatological and oral adverse events

Category	Intervention	Mechanism	Outcomes	References
**Clinical Presentation and Initial Management**	NSAIDs and/or analgesics	• Reduce inflammation and pain through cyclooxygenase inhibition.	• Effective for mild-to-moderate rheumatic irAEs, but low efficacy (< 60%) as monotherapy, necessitating additional treatments.	[189–192]
	Systemic glucocorticoids	• Suppress immune response by inhibiting inflammatory cytokines.	• Symptom control in nearly all cases, used in ~ 2/3 of patients.	[193–195]
	csDMARDs (e.g., MTX, hydroxychloroquine, sulfasalazine)	• Modulate immune response, targeting specific inflammatory pathways.	• Symptom control in ~ 90% of cases, enables glucocorticoid tapering.	[189, 196, 197]
	bDMARDs (e.g., TNF inhibitors, tocilizumab)	• Target specific cytokines (TNF, IL-6) to reduce inflammation.	• Effective in ~ 10% of patients, particularly for glucocorticoid non-responders.	[198]
**Glucocorticoid Therapy**	Systemic glucocorticoids (10–20 mg/day prednisone, up to 40 mg/day for severe cases)	• Inhibit pro-inflammatory cytokine production and immune cell activation.	• High response rate, symptom control in most cases, but prolonged use may risk tumor response interference.	[81, 119, 195]
	Intra-articular glucocorticoid injections	• Local anti-inflammatory effect in affected joints.	• Effective in ~ 15% of patients with mono- or oligoarthritis, favorable local response.	[81]
**csDMARD and bDMARD Therapy**	MTX (10–20 mg/week)	• Inhibits folate metabolism, reducing immune cell proliferation.	• Promising for polyarthritis, supports glucocorticoid sparing, minimal impact on ICI efficacy.	[197, 204, 207]
	TNF inhibitors	• Block TNF-alpha to reduce inflammation.	• Effective in 70% of bDMARD-treated patients for steroid-refractory arthritis.	[24, 198, 207]
	Tocilizumab	• Inhibits IL-6 receptor, reducing systemic inflammation.	• Effective in patients with inadequate response to TNF inhibitors.	[24, 198]
	JAK inhibitors (e.g., tofacitinib)	• Inhibit JAK-STAT signaling, modulating immune response.	• Emerging option, efficacy on ICI response not fully established.	[205]
**Optimal Therapeutic Strategies**	NSAIDs + intra-articular corticosteroids (Grade 1)	• Local and systemic anti-inflammatory effects with minimal immunosuppression.	• Alleviates mild symptoms, but limited by GI, renal, and cardiovascular risks.	[199, 202]
	Systemic glucocorticoids (10–20 mg/day, Grade 2)	• Broad immunosuppression to control joint inflammation.	• Effective for moderate arthritis, tapered over weeks/months based on response.	[203]
	High-dose glucocorticoids (0.5–1 mg/kg/day, Grade 3–4)	• Aggressive immunosuppression for severe cases.	• Rapid symptom control, tapered quickly to minimize immunosuppression duration.	[24, 87]
	DMARDs (MTX, leflunomide, sulfasalazine)	• Target specific immune pathways to control chronic inflammation.	• Reduces inflammation, prevents joint damage, supports glucocorticoid sparing.	[87, 204]
**Discontinuation and Reintroduction**	Temporary ICI discontinuation	• Pause ICI to manage severe irAEs (Grade 3–4).	• Required in ~ 25% of patients; most resume ICI once arthritis is controlled.	[81, 209]
	Reintroduction post-control	• Resume ICI after irAE resolution, balancing cancer and irAE management.	• Feasible in most non-life-threatening cases, guided by multidisciplinary assessment.	[209]
**Management of Oral Disorders**	Topical corticosteroids	• Local anti-inflammatory effect on oral mucosa.	• Reduces inflammation in oral mucositis and lichenoid reactions.	[15]
	Analgesics (e.g., transdermal fentanyl)	• Pain relief through opioid receptor agonism.	• Effective for severe mucositis pain management.	[15]
	Topical antifungals	• Prevent secondary fungal infections in immunocompromised patients.	• Reduces risk of oral candidiasis.	[15]
	Photobiomodulation (low-level lasers)	• Stimulates tissue repair, reduces inflammation.	• Effective for mucositis, xerostomia, and dysgeusia.	[15]
	Saliva stimulants (pilocarpine, cevimeline)	• Stimulate salivary gland function.	• Alleviates xerostomia, reduces dental complications.	[15]
	Oral zinc supplements	• Supports taste bud function and mucosal repair.	• Improves dysgeusia in < 3% of patients.	[15]
	Preoperative oral care (teeth cleaning, scaling)	• Reduces oral bacterial load and inflammation.	• Lower postoperative CRP levels (5.69 vs. 7.90 mg/dl, *P* = 0.030), reduced complications.	[214]
	Antibiotic prophylaxis (e.g., cefazolin, doxycycline)	• Targets pathogens like Staphylococcus aureus, reduces infection risk.	• Mitigates infection risk in immunocompromised patients or severe oral irAEs.	[15, 216]
	PJP prophylaxis (sulfamethoxazole/trimethoprim)	• Prevents opportunistic infections in prolonged steroid use.	• 43% infection rate despite prophylaxis, effectiveness questioned.	[215]

A collaborative approach is essential, with oral healthcare providers making significant contributions to diagnosing and managing oral irAEs through clinical examination, patient history, and targeted treatments. Oncologists and specialists collaborate to integrate cancer therapy with oral health, ensuring comprehensive patient care. Regular dental examinations and patient education on oral hygiene are essential for preventing complications, especially in patients at risk for infections due to immunosuppression [15]. This multidisciplinary approach ensures the integration of cancer treatment and oral health, although specific guidelines for ICI patients are still under development [134].

## Drug interactions

ICIs, which are rapidly being utilized across a diverse patient population, including those who may be receiving concurrent antirheumatic or dental management pharmacotherapies, present critical considerations regarding drug interactions. Given that their immunomodulatory mechanism primarily targets immune checkpoints (PD-1, PD-L1, and CTLA-4), interactions may arise with any drug that affects inflammatory or immune pathways [217].

Patients with autoimmune diseases or those requiring antirheumatic therapy represent a unique group, as they may already be on immunosuppressive medications such as corticosteroids, MTX, or biologics (e.g., TNF-α inhibitors, IL-6 inhibitors) [218]. These agents can suppress immune responses, potentially limiting the effectiveness of ICIs. Clinical data suggest that patients receiving high-dose corticosteroids at the initiation of ICI therapy exhibit poorer outcomes, likely due to diminished T-cell activation. Evaluation of the effects of the lowest doses of dexamethasone and prednisone on various aspects of T cell activity in an ICI setting showed that prednisone, at physiological concentrations, did not affect T cell cytokine (IL-2, IFN-γ, TNF-α) production or primary T cell proliferation in vivo, even when administered in combination with anti-PD-1/CTLA-4 antibodies [219]. It also did not impact co-inhibitory receptor expression (PD-1, CTLA-4, TIM-3, LAG-3) and decreased LAG-3 expression, while dexamethasone downregulated LAG-3. Co-treatment of PD-1^+^ Jurkat cells with prednisone and dexamethasone with anti-PD-1 decreased SHP-2 phosphorylation, indicating enhanced T-cell function [219]. These findings suggest that prednisone does not impair ICI efficacy and, therefore, should be approached differently from dexamethasone in immunotherapy management [220]. Hence, clinicians should carefully consider disease control for autoimmune disorders against the potential impact on ICI therapy, ideally in a multidisciplinary manner.

Due to the protocols used with specific drugs during dental practices, dental medications, especially antibiotics and drugs aimed at controlling inflammation, should also be considered [221]. Common antibiotics used in dental settings (e.g., amoxicillin-clavulanate or metronidazole) can disrupt the gut microbiome [222]. As discussed, changes in the gut microbiota (dysbiosis) can influence systemic immune responses and, in turn, affect ICI efficacy [223]. While short-term antibiotic use has not demonstrated a clinical impact, ongoing investigations suggest that cautious prescription should be advised during ICI therapy. Furthermore, common NSAIDs used for dental pain management could influence ICI treatment by modulating prostaglandin pathways, although clear evidence linking NSAID use with ICI responsiveness is currently lacking [224]. Dentists treating patients undergoing ICI therapy should be aware of these potential issues and collaborate closely with oncologists to minimize any negative impacts on treatment outcomes.

Addressing drug interactions is vital for optimizing ICI therapy. Effective interdisciplinary communication, coupled with well-coordinated patient management at an integrated level, is crucial for addressing the known interactions of ICIs with antirheumatic and dental medications. Further research is necessary to clarify the scope and clinical significance of these interactions, enabling patients to receive the most effective and safest care possible.

## Biomarkers for immune-related adverse events

Most irAEs are mild and reversible when identified early and managed appropriately; thus, biomarkers for predicting their occurrence are crucial [225]. Compared to biomarkers for tumor response, biomarkers for irAEs have been less extensively studied, with some identified irAE biomarkers showing overlap with those related to tumor responses. Several biomarkers associated with irAEs in cancer patients receiving treatment with ipilimumab, nivolumab, and atezolizumab have been reported in several studies [225]. Body composition parameters, such as sarcopenia and reduced muscle area, have been recognized as independent factors associated with high-grade irAEs in melanoma patients [226]. Sex differences indicated that females with melanoma had higher rates of irAEs [227], while increased baseline IL-6 levels were associated with colitis-related irAEs [228]. Elevations in circulating IL-6 and IL-17 following treatment were significantly correlated with irAEs in melanoma patients [228, 229]. Soluble CD163, CXCL5, and blood cell counts, including absolute lymphocyte and eosinophil counts, were identified as predictors of irAEs in melanoma, renal cell carcinoma (RCC), and urothelial carcinoma [230, 231]. Autoantibodies, including anti-PD-1 and thyroid-stimulating hormone (TSH)/thyroid peroxidase (TPO) antibodies, have been associated with endocrine and thyroid irAEs in melanoma and NSCLC patients [228]. T cell repertoire diversity and gut microbiome composition, specifically the enrichment of *Faecalibacterium* and *Bacteroidetes*, were correlated with the incidence and resistance of irAEs [232, 233]. In RA, PD-1 expression on peripheral T cells is reduced and inversely correlates with disease activity, whereas synovial T cells exhibit high but functionally impaired PD-1 expression, which may contribute to inflammation [234, 235]. The levels of sPD-1 are inconsistent, with studies reporting elevated levels correlating with disease activity and reduced levels in RA patients [236, 237]. In vitro, sPD-1 promotes the secretion of pro-inflammatory cytokines and cell proliferation [238]. Further research is needed to resolve these discrepancies and establish sPD-1 as a reliable biomarker for RA.

## Concluding remarks & future perspectives

This review elucidates the prevalent immunopathological mechanisms implicated in ICI-induced arthritis and oral disorders, which are affected by dysregulated T-cell responses, pro-inflammatory cytokines (including IL-6, TNF-α, and IFN-γ), and the reciprocal interactions noted in the correlations between RA and PERIODONTAL DISEASE. The principal findings indicate that the incidence of ICI-IA varies from 4% to 6%, escalating to 43% in combination therapy, whereas oral irAEs occur at a rate of 5% to 7%. These illnesses demonstrate clinical parallels to autoimmune disorders such as RA and Sjögren’s syndrome, with a possibility for chronicity noted in 25% of arthritis cases after the discontinuation of immune checkpoint inhibitors. irAEs diminish quality of life while potentially correlating with improved tumor responses, highlighting the intricate relationship between therapy efficacy and toxicity.

Notwithstanding these insights, significant gaps persist, including the inadequately examined interplay between arthritis and oral disorders in relation to ICIs, an absence of diagnostic biomarkers for the early prediction of irAEs, and insufficient data concerning long-term outcomes following the discontinuation of ICIs. The lack of cohesive interdisciplinary protocols hinders personalized management, particularly in differentiating irAEs from the effects of chemotherapy or pre-existing diseases.

Future research must focus on bridging these gaps by prospective studies on cytokine profiles and microbial impacts, including Porphyromonas gingivalis citrullination, to clarify the arthritis-oral feedback loop. The following are recommendations for the research agenda: (1) The development of biomarkers, such as the assessment of synovial fluid white blood cell counts or PD-1^+^ tissue-resident memory T cells, to predict the severity and phenotypes of immune checkpoint inhibitor-related inflammatory arthritis [115]; (2) The execution of interventional studies, including randomized controlled trials, to evaluate IL-6 inhibitors (e.g., tocilizumab) for steroid-refractory irAEs or prophylactic oral care regimens aimed at mitigating mucositis and xerostomia; and (3) The implementation of longitudinal cohort studies to examine the effects of early immunosuppression on anti-tumor efficacy [239].

We advise prompt referral to a rheumatologist and oral health specialists upon suspicion of irAEs, in alignment with revised guidelines, such as the NCCN 2024 guidelines, for multidisciplinary management of toxicity. These guidelines emphasize the importance of immediate intervention with corticosteroids or biologics while monitoring for infections [240]. Prophylactic measures, such as initial oral evaluations and hydration procedures for oral irAEs, may avert the necessity for intravenous support or treatment delays. These developments will enhance the efficacy of ICI, hence enhancing patient outcomes in oncology and diminishing rheumatologic and oral morbidity.

## Data Availability

Not applicable.
